# Supplementary material for: Optimising age coverage of seasonal influenza vaccination in England: A mathematical and health economic evaluation
Source: PLoS Comput Biol. 2020 Oct 6;16(10):e1008278. doi: 10.1371/journal.pcbi.1008278 (PMC7567368; doi:10.1371/journal.pcbi.1008278)
Supplement: S1 Text — This supplement consists of: (1) Data descriptions; (2) Complementary details of the epidemiological modelling approach; (3) Complementary details of the health economic modelling approach; (4) Additional results. (PDF) [file pcbi.1008278.s001.pdf]

Supporting Information
Optimising age coverage of seasonal influenza vaccination in England: A mathematical and health economic evaluation

Edward M. Hill[1*], Stavros Petrou[2,3], Henry Forster[4], Simon de Lusignan[3,5], Ivelina Yonova[3,5], Matt J. Keeling[1]

1 The Zeeman Institute for Systems Biology & Infectious Disease Epidemiology Research, School of Life Sciences and Mathematics Institute, University of Warwick, Coventry, CV4 7AL, United Kingdom.
2 Warwick Clinical Trials Unit, Warwick Medical School, University of Warwick, Coventry, CV4 7AL, United Kingdom.
3 Nuffield Department of Primary Care Health Sciences, University of Oxford, Oxford, OX2 6GG, United Kingdom.
4 Government Statistics Service, Department of Health and Social Care, Leeds, LS2 7UE, United Kingdom.
5 Royal College of General Practitioners, London, NW1 2FB, United Kingdom.

* Corresponding Author. Email: Edward.Hill@warwick.ac.uk

Table of Contents

1 Data descriptions **2**
1.1 Consultations in General Practices 2
1.2 Respiratory Virus RCGP Surveillance 3
1.3 Circulating strain composition 3
1.4 Vaccine uptake 4
1.5 Vaccine efficacy 5

2 Complementary details of the epidemiological modelling approach **9**
2.1 Model framework schematic 9
2.2 Immunity propagation model: Susceptibility array 9
2.3 Epidemiological model specifics 10
2.3.1 Contact matrix 11
2.3.2 Between-season exposure history group mappings 12
2.3.3 End of influenza season demographic processes 12

3 Complementary details of the health economic modelling approach **13**
3.1 Recovering risk group specific GP consultation rates 13
3.2 Hospital Episode Statistics: Data extract overview 13
3.2.1 The data extracts 13
3.2.2 Determining risk status 14
3.2.3 Emergency admissions 14
3.2.4 Death events 15

3.2.5 Outpatient attendance . . . 15
3.2.6 Omitted cost elements . . . 15
3.3 Clinical outcome assumptions by influenza type . . . 16
3.4 Clinical monetary costs by influenza type . . . 18
3.5 Fatal influenza case QALY loss calculation . . . 19

4 Additional results . . . 20
4.1 Model fitting . . . 20
4.2 Model goodness-of-fit . . . 21
4.3 Simulating vaccination programmes . . . 22

1 Data descriptions

1.1 Consultations in General Practices

The text contained in this subsection was provided by the Royal College of General Practitioners (RCGP). Thus, throughout this subsection "we" refers to contributors from RCGP who extracted the data.

Method:
Data were extracted from four RCGP Research and Surveillance Centre (RSC) databases [1]. UK general practice is a registration based system where all citizens can register with a single GP of their choice. Practices are computerised, and data entered into computerised medical record systems either as coded data [2], or free text. We extracted the coded data, and our results are based on this element of the record [3]. We extract all coded data, pseudonymising as close to sources as possible. Where patients have a range of codes inserted suggesting they opt out of record sharing we do not analyse their data [4].

The data sources was the Real World Evidence (RWE) database, established at University of Surrey in March 2015. This database contains all continuous historical data contained in the GP systems. Bulk data are extracted four times per year taking historic coded data. Where extracts fail for major reports we attempt to extract data using Morbidity Information and Export Syntax (MIQUEST). This is a Department of Health sponsored data extract tool.

RCGP provided weekly influenza-like-illness (ILI) rates for the RSC population from week 1 2000 to week 52 2018, disaggregated as follows:

1. **ISOYear**
2. **ISOWeek**
3. **Age**
4. **Chronic Disease**
5. **Population**
6. **Number of people with ILI**
7. **Rates per 100,000**

The data compared the ILI rates of people with chronic diseases (Chronic Disease = 1) against people without chronic diseases (Chronic Disease = 0).

Contributors:

1. **Simon de Lusignan** – Director, guarantor for these data, assisted with clinical knowledge
2. **Rachel Byford** – Put together structures for the data extraction.
3. **Ana Correa** – Carried out the quality assessment of the output.
4. **Chris McGee** – Carried out data extraction of ILI rates (covering week 1 2000 to week 52 2016).
5. **Julian Sherlock** – Carried out data extraction of ILI rates and data quality checks (covering week 1 2017 to week 35 2018).
6. **Sameera Pathirannehelage** – Carried out data extraction of ILI rates (covering week 1 2017 to week 52 2018).
7. **Ivelina Yonova** – liaison with practices, and review of the original data request.

Acknowledgements:
Practices who have agreed to be part of the RCGP RSC and allow us to extract and used health data for surveillance and research. Filipa Ferreira (programme manager), and other members of the Clinical Informatics and Health Outcomes Research Group at University of Surrey. Apollo Medical Systems for data extraction. Collaboration with EMIS, TPP, In-Practice and Micro-test CMR supplier for facilitating data extraction. Colleagues at Public Health England.

1.2 Respiratory Virus RCGP Surveillance

We sourced data pertaining to the weekly percentage of sentinel virology samples that were influenza positive from a subset of general practices in the RCGP Weekly Returns Service, which submitted respiratory samples for virological testing from patients presenting in primary care with an ILI.

These data were displayed in figures within PHE annual influenza reports (for the 2009/10 to 2017/18 influenza seasons inclusive) [5, 6], and PHE Weekly National Influenza reports (for the 2013/14 influenza season onwards) [7].

PHE annual influenza reports were our baseline source for virological sample positivity data. Typically, curves displaying the data covered week 40 up to a variable week number dependent upon the influenza season (week 13: 2016/17; week 14: 2010/11; week 15: 2011/12, 2014/15, 2017/18; week 20: 2013/14). As an exception, the data curve for the entire 2009/10 influenza season was available within the 2010/11 PHE annual influenza report [5].

In all influenza seasons, we assumed weeks 36-39 and weeks 21-39 had an influenza positivity of 0%.

To inform positivity values for weeks preceding week 20 for the 2013/14 influenza season onwards that were not illustrated within the PHE annual influenza report, we used PHE Weekly National Influenza reports [7], which provided United Kingdom GP sentinel swabbing scheme sample positivity on a weekly basis. Note that we gave precedence to figures explicitly stated within annual reports over positivity quantities within the weekly PHE influenza summaries.

1.3 Circulating strain composition

Here, we expand on our use of FluNet [8] to inform the circulating influenza strain distribution per influenza season.

FluNet is a global web-based tool for influenza virological surveillance first launched in 1997. The virological data entered into FluNet, e.g. number of influenza viruses detected by subtype, are critical for tracking the movement of viruses globally and interpreting the epidemiological data.

FluNet reports weekly influenza surveillance data that are provided from over 140 National Influenza Centres of the Global Influenza Surveillance and Response System, national influenza reference laboratories, and WHO regional databases [9]. Per the WHO Global Epidemiological Surveillance Standards for Influenza guidance, influenza testing is conducted on specimens collected from persons presenting for medical care at participating surveillance sites who meet a clinical definition for influenza-like illness (defined as an acute respiratory infection with measured fever of $\geq 38°$C, and cough, with onset within the last 10 days), or severe acute respiratory infection (defined as an acute respiratory infection with history of fever or measured fever of $\geq$ 38°C, and cough, with onset within the last 10 days, and requiring hospitalization) [9]. Influenza is confirmed by accepted laboratory diagnostic methods and actively reported by the reference laboratory to FluNet [10].

We computed the empirical circulating strain distribution in each influenza season (from the 2009/10 influenza season onward) using data from FluNet for the United Kingdom. A salient feature of the influenza type data is the presence of samples whose exact subtype/lineage were not determined (Fig. A). We assumed the fraction of undetermined samples ascribed to each subtype/lineage matched the proportions observed for the set of samples where strain-specific information were available (Fig. B)

1.4 Vaccine uptake

For the 2009/10 influenza season, we acquired vaccine uptake profiles from survey results on H1N1 vaccine uptake amongst patient groups in primary care [11].

For the 2010/11 influenza season onward, we sourced seasonal influenza vaccine uptake figures from Public Health England official statistics [6, 7]. Taken from PHE Weekly National Influenza Reports, these data comprised uptake profiles per target vaccination age group (e.g. at-risk under 65 years, 65 year and over, 2 year-olds, 3 year-olds) for each influenza season.

We constructed daily uptake rates from either weekly or monthly uptake values via linear interpolation. The temporal resolution of the empirical vaccine uptake data was dependent upon the age category, stratified by: (i) non-school aged children (weekly coverage figures); (ii) school-aged children (monthly coverage figures).

(i) Non-school aged children Delivery of vaccination to those aged under 65 years in a clinical risk group or 65+ years old is through their GP. Additionally, all 2 and 3 year-olds eligible for influenza vaccination are administered the vaccine through their GPs. Uptake profiles are constructed based upon GP practices reporting weekly to Immform (the influenza vaccine uptake monitoring programme from Public Health England).

For the groupings of at-risk under 65 years and 65 years and above, vaccine uptake profiles were available from 2012/13 influenza season onwards. For the 2010/11 and 2011/12 influenza seasons we invoked the uptake profile from the 2012/13 influenza season.

Collection of cumulative weekly vaccine uptake for 2 year-olds and 3 year-olds started in 2013/14.

(ii) School-aged children For school-aged children, monthly vaccination uptake estimates were available. These uptake estimates specified the proportion of children in England who received the influenza vaccine via school, pharmacy or GP practice.

(a)

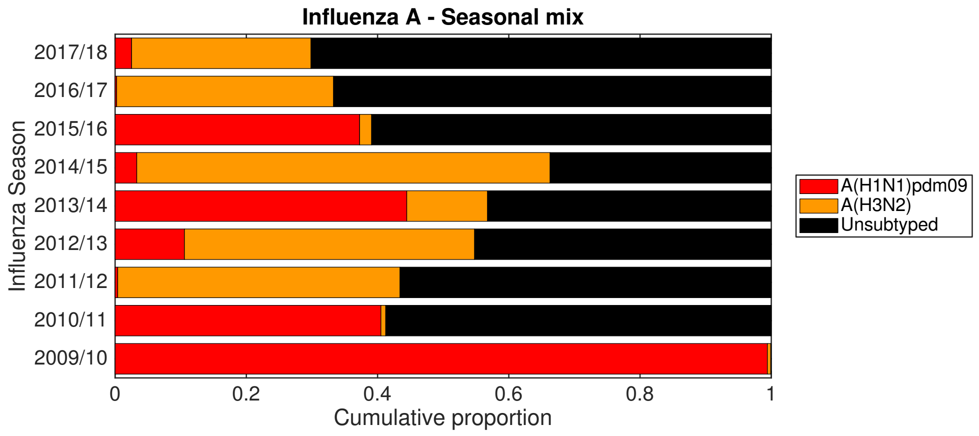

(b)

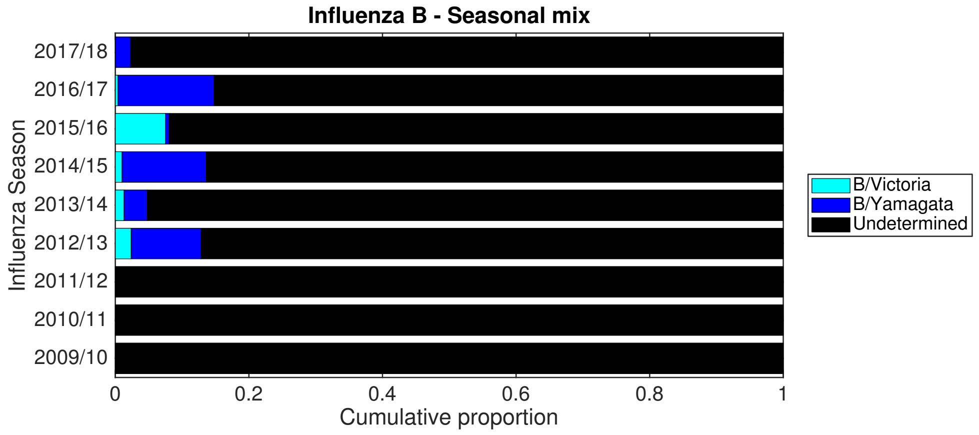

Fig A. Breakdown of subtype/lineage composition of sampled influenza viruses, per influenza type, from the United Kingdom in each influenza season (2009/10 onward). (a) Type A influenza, stratified by subtype; **(b)** Type B influenza, stratified by lineage.

1.5 Vaccine efficacy

We informed age dependent vaccine efficacy estimates for each historical influenza season from publications detailing end-of-season age adjusted seasonal influenza vaccine effectiveness for adults and children in preventing laboratory-confirmed influenza in primary care in the United Kingdom [12–18]. In influenza seasons where an equivalent publication were not available, we used mid-season or provisional end-of-season age adjusted seasonal vaccine efficacy estimates from Public Health England reports [19, 20].

The 2009/10 influenza season was an exception. Due to the adjusted seasonal influenza vaccine efficacy being -30% (-89%, 11%) [12], we instead used the pandemic vaccine to inform efficacy against A(H1N1)pdm09, with the effectiveness against all other types set to zero.

The age- and strain-specific adjusted vaccine efficacy estimates gathered from the literature are displayed in Table A. In the United Kingdom, since the incremental introduction of the universal childhood influenza vaccine programme began in the 2013/14 influenza season, an intra-nasally administered live attenuated influenza vaccine (LAIV) has been administered to children. Adult age classes have generally been offered an inactivated influenza vaccine (IIV). Therefore, from

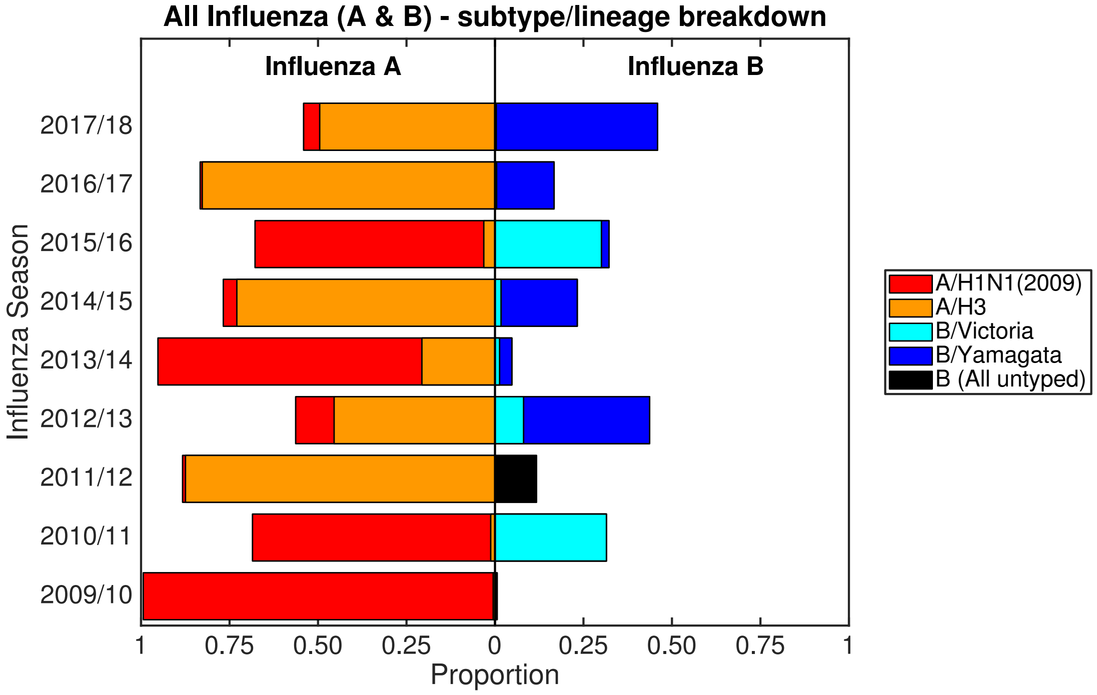

Fig B. Breakdown of subtype/lineage composition of sampled influenza viruses, as a proportion of all influenza samples, from the United Kingdom in each influenza season (2009/10 onward). Sequenced samples confirmed as influenza positive, stratified by type and subtype/lineage. We assumed the fraction of undetermined samples ascribed to each subtype/lineage matched the observed proportions (for influenza seasons where strain-specific information were available).

that time point efficacy estimates applicable to children and adults correspond to IIV and LAIV effectiveness, respectively.

With the empirical data not providing individual estimates for each influenza A subtype and influenza B lineage, we invoked a series of assumptions to produce the strain-specific vaccine efficacy quantities used within our study (Table 1).

We outline herein the set of enacted assumptions.

Approach to address absent influenza A efficacy data

Case one: Data absent for a single subtype only

Applicable to the following influenza seasons: 2014/15, 2016/17.

When there were estimates present for overall influenza A efficacy, but estimates for only one of the A(H1N1)pdm09 or A(H3N2) subtypes, for the subtype with no efficacy data we assumed its efficacy matched the overall influenza A efficacy estimate.

Case two: Data absent for one subtype and overall influenza A efficacy

Applicable to the following influenza seasons: 2010/11, 2011/12, 2015/16.

In those influenza seasons where there was an efficacy estimate for one of the two influenza A subtypes, but efficacy estimates for the other influenza A subtype and against influenza A overall were absent, we assumed the efficacy for the subtype with no relevant empirical data

available was equal to that of the other influenza A subtype (for which there was an available estimate).

Case three: Age-group specific estimates - sample numbers too low

Applicable to the 2012/13 influenza season only.

When the number of samples for an age group and influenza type combination were too small to allow for a vaccine efficacy estimate to be made, we instead used the overall population vaccine efficacy estimate against that particular strain type.

Approach to address absent influenza B efficacy data

Applicable to all influenza seasons from 2011/12 up to and including 2017/18.

When there were estimates present for overall influenza B efficacy, together with estimates for only one of the B/Yamagata and B/Victoria lineages, for the subtype with no efficacy data we assumed its efficacy matched the overall influenza B efficacy estimate.

Approach to populate efficacy estimates for those aged under two

Beginning with the 2014/15 influenza season, age banded efficacy estimates were provided for those aged two and above. Therefore, in those latter influenza seasons there was an absence of estimates for those younger than two years of age. Given those under two years of age would receive a trivalent vaccine, we set the vaccine efficacy for the under two years of age class to match vaccine efficacies for either the 18–44 age group (applied to the 2014/15 and 2015/16 influenza seasons) or the 18–64 age group (2016/17 and 2017/18 influenza seasons).

Table A. Adjusted influenza vaccine effectiveness estimates by influenza season, age and strain type. 95% confidence intervals are stated within parentheses.

Influenza season	Age	VE % (95% CI)						Sour
		A(H1N1)pdm09	A(H3N2)	All A	B/Vic	B/Yam	All B	
2009/10	All	72 (21, 90)	0	—	0	0	0	[12]*
2010/11	0–4	87 (45, 97)	—	—	47 (-337, 93)	N/A	47 (-337, 93)	[13]
	5–14	84 (27, 97)	—	—	75 (32, 91)	N/A	75 (32, 91)	
	15–44	46 (29, 71)	—	—	61 (35, 78)	N/A	61 (35, 78)	
	45–64	52 (27, 67)	—	—	45 (10, 68)	N/A	45 (10, 68)	
	65+	74 (1, 88)	—	—	67 (44, 86)	N/A	67 (44, 86)	
2011/12	0–4	—	52 (-446, 96)	—	—	—	—	[14]
	5–14	—	69 (-172, 97)	—	—	—	—	
	15–44	—	7 (-67, 48)	—	—	—	—	
	45–64	—	11 (-56, 49)	—	—	—	—	
	65+	—	48 (-50, 82)	—	—	—	—	
2012/13	0–4	n too low	n too low	n too low	—	—	n too low	[15]

	5–14	n too low	n too low	n too low	—	—	74 (1, 93)	
	15–44	83 (28, 96)	40 (-7, 66)	54 (21, 73)	—	—	68 (46, 82)	
	45–64	90 (20, 99)	32 (-27, 63)	37 (-10, 63)	—	—	34 (-1, 57)	
	65+	n too low	-14 (-206, 57)	-19 (-44, 47)	—	—	65 (18, 85)	
2013/14	All	—	—	61 (-8, 86)	—	—	61 (-8, 86)	[19]†
2014/15	2–17	—	29.4 (5.8, 47.1)	30.4 (8.4, 47.2)	—	—	59.4 (-48.1, 88.8)	[16]
	18–44	—	30.3 (-12.4, 56.7)	34.5 (-3.0, 58.4)	—	—	40.4 (-50.9, 76.5)	
	45–64	—	31.1 (-5.8, 55.2)	32.4 (-1.8, 55.2)	—	—	49.2 (-0.4, 74.3)	
	65+	—	32.6 (-44.5, 68.6)	30.2 (-46.4, 66.7)	—	—	-203 (-2,300, 61.7)	
2015/16	2–17	48.5 (8.5, 71.0)	—	—	—	—	76.5 (41.9, 90.5)	[17]
	18–44	59.8 (35.8, 74.8)	—	—	—	—	45.9 (1.0, 70.4)	
	45–64	58.6 (36.9, 72.8)	—	—	—	—	65.0 (15.1, 85.6)	
	65+	56.1 (7.2, 79.3)	—	—	—	—	-20.2 (-259.1, 59.8)	
2016/17	2–17	—	57.0 (7.7, 80.0)	63.3 (22.0, 82.7)	—	—	78.6 (-86.0, 97.5)	[18]
	18–64	—	36.6 (10.4, 55.1)	38.5 (15.1, 55.3)	—	—	52.1 (-20.0, 80.9)	
	65+	—	-68.4 (-248.9, 18.7)	-21.2 (-134.4, 37.3)	—	—	17.2 (-249.7, 80.4)	
2017/18	2–17	90.3 (16.4, 98.9)	-75.5 (-289.6.7, 21.0)	—	—	—	60.8 (8.2, 83.3)	[21]‡
	18–64	69.1 (11.4, 89.2)	-14.7 (-72.7, 23.8)	—	—	—	18.2 (-15.1, 41.9)	
	65+	—	16.8 (-74.2, 60.3)	—	—	—	13.2 (-68.4, 55.2)	

Data not specified denoted by —.
For influenza seasons where TIV were in use (rather than QIV), N/A in an influenza B field corresponds to the lineage absent from the TIV composition and no efficacy estimate was given (we assume to be 0%).
*: The adjusted seasonal influenza vaccine efficacy in the 2009/10 influenza season was -30% (-89%, 11%) [12]. We therefore used the pandemic vaccine to inform efficacy against A(H1N1)pdm09, with the effectiveness against all other types set to zero.
†: Mid-season estimate of seasonal influenza vaccine effectiveness from [19]. Low incidence throughout the 2013/14 influenza season meant reliable end-of-season estimates for the vaccine efficacy could not be attained (Personal communication, Public Health England).
‡: Provisional end-of-season influenza vaccine effectiveness results.

2 Complementary details of the epidemiological modelling approach

2.1 Model framework schematic

We provide below a visual depiction of the entire model structure and incorporation of the data streams within and between the four model components: vaccination model, immunity propagation model, epidemiological model and observation model (Fig. C).

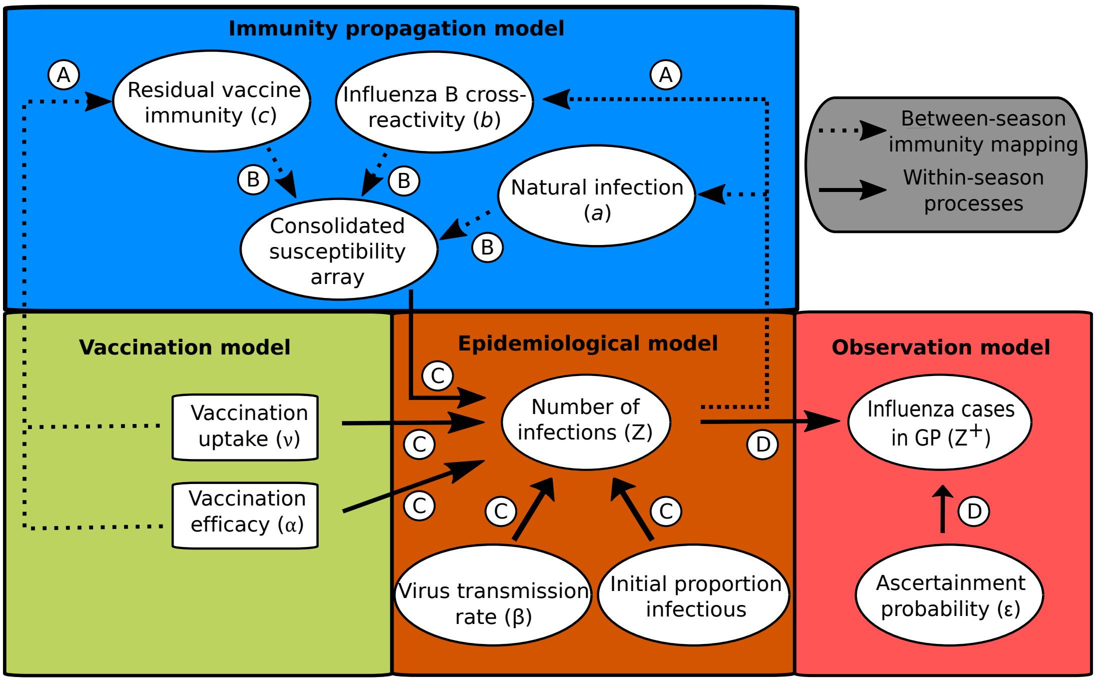

Fig C. Schematic showing the links between the vaccination, immunity propagation, epidemiological and observation model components. We adopt the visualisation conventions of [22], with ellipses indicating variables, and rectangles indicating data. Dotted arrows indicate relationships between prior season epidemiological outcomes and immunity propagation factors. Solid arrows indicate within-season processes. Circled capitalised letters indicate the relationships connecting the variables or data involved. These relationships are: process A, propagation of immunity as a result of exposure to influenza virus in the previous influenza season (through natural infection or vaccination); process B, modulation of current influenza season virus susceptibility; process C, estimation of influenza case load via the SEIR model of transmission; process D, ascertainment of cases through ILI recording at GP. Reproduced from Hill *et al.* [23].

2.2 Immunity propagation model: Susceptibility array

We had a total of ten exposure history groupings and associated strain-specific susceptibilities, which we consolidated into a single susceptibility array (Fig. D).

We expand here our protocol for parameterisation of susceptibility values amongst exposure groups capturing those both vaccinated and naturally infected in the prior season (Fig. D: rows 7–10).

For this collection of exposure histories, susceptibility to a subset of strains may conceivably be collectively modified via natural infection and vaccination immunity propagation pathways. In these instances, we treated the immunity propagation mechanisms independently, with the modified susceptibility set by the dominant immunity propagation entity (i.e. $\min(a, c_m)$, $\min(b, c_m)$).

Exposure history (h)		Strain susceptibility			
		A(H1N1)pdm09	A(H3N2)	B/Victoria	B/Yamagata
	Naïve	1	1	1	1
	A(H1N1)pdm09	a	1	1	1
	A(H3N2)	1	a	1	1
	B/Victoria	1	1	a	b
	B/Yamagata	1	1	b	a
	Vacc. (V)	$c_{A(H1N1)}$	$c_{A(H3N2)}$	$c_{B/Victoria}$	$c_{B/Yamagata}$
	A(H1N1)pdm09 & V	$\min(a, c_{A(H1N1)})$	$c_{A(H3N2)}$	$c_{B/Victoria}$	$c_{B/Yamagata}$
	A(H3N2) & V	$c_{A(H1N1)}$	$\min(a, c_{A(H3N2)})$	$c_{B/Victoria}$	$c_{B/Yamagata}$
	B/Victoria & V	$c_{A(H1N1)}$	$c_{A(H3N2)}$	$\min(a, c_{B/Victoria})$	$\min(b, c_{B/Yamagata})$
	B/Yamagata & V	$c_{A(H1N1)}$	$c_{A(H3N2)}$	$\min(b, c_{B/Victoria})$	$\min(a, c_{B/Yamagata})$

Fig D. Infographic presenting the interaction between exposure history and susceptibility. The interaction between exposure history h and susceptibility to strain m in the current season, $f(h,m)$, was classified into ten distinct groups: One group for the naive (uninfected and not vaccinated, row 1); one group per strain, infected but not vaccinated (rows 2–5); one group for those vaccinated and experiencing no natural infection (row 6); one group per strain for being infected and vaccinated (rows 7–10). We let a denote modified susceptibility to strain m given infection by a strain m type virus the previous season (dark green shading), b modified susceptibility due to cross-reactivity between type B influenza lineages (dark blue shading), and c_m the change in susceptibility to strain m given vaccination in the previous season (gold shading). Unmodified susceptibilities retained a value of 1 (red shading). We enforced $0 < a,b,c_m < 1$. Reproduced from Hill *et al.* [23].

In other words, we took a pessimistic stance by assuming no boosting of the immunity propagation response as a result of dual influenza virus exposure (from both natural infection and vaccination).

2.3 Epidemiological model specifics

The epidemiological model was a deterministic, age-dependent (single year age brackets), multi-strain structured compartmental based model capturing influenza infection status (with susceptible-latent-infected-recovered, SEIR, dynamics) and vaccine uptake. In addition, we assumed disease transmission to be frequency-dependent.

We let $E_i^{X,m}$, $I_i^{X,m}$ and $R_i^{X,m}$ denote the proportion of the population that were in age class i, with vaccination status $X \in \{N, V\}$, and that were latent, infectious and recovered (as a result of natural infection) with respect to strain m.

Exposure to influenza virus in the previous influenza season, through natural infection or vaccination, modulated current influenza season susceptibility. Tracking immunity derived from natural infection and vaccination separately required, per age bracket, ten distinct exposure history groups, with the susceptibility to strain m for a given exposure history group h encoded into the susceptibility array $f(h,m)$.

To incorporate exposure history from the previous influenza season, we let $S_i^{X,h}$ denote the proportion of the population that are in age class i, with exposure history h and vaccination status $X \in \{N, V\}$ that are susceptible to all strains. With ten exposure history groups h in place, a total of 20 susceptibility states were used: ten $S^{N,h}$ states, accounting for susceptibles not vaccinated in the current influenza season stratified by exposure history grouping; ten $S^{V,h}$

states, tracking susceptibles who had been administered the vaccine in the current influenza season whilst retaining exposure history group information.

The epidemiological model had the following formulation, with time dependencies dropped:

$$\begin{cases} \frac{dS_i^N}{dt} &= -\left(\sum_m \left(\sum_h f(h,m) S_i^{N,h}\right) \lambda_i^m\right) - \mu_i S_i^N \\ \frac{dS_i^V}{dt} &= -\left(\sum_m \left(\sum_h f(h,m) S_i^{V,h}\right) (1-\alpha_i^m)\lambda_i^m\right) + \mu_i S_i^N \\ \frac{dE_i^{N,m}}{dt} &= \left(\sum_h f(h,m) S_i^{N,h}\right) \lambda_i^m - \gamma_1^m E_i^{N,m} - \mu_i E_i^{N,m} \\ \frac{dI_i^{N,m}}{dt} &= \gamma_1^m E_i^{N,m} - \gamma_2 I_i^{N,m} - \mu_i I_i^{N,m} \\ \frac{dR_i^{N,m}}{dt} &= \gamma_2 I_i^{N,m} - \mu_i R_i^{N,m} \\ \frac{dE_i^{V,m}}{dt} &= \left(\sum_h f(h,m) S_i^{V,h}\right) (1-\alpha_i^m)\lambda_i^m - \gamma_1^m E_i^{V,m} + \mu_i E_i^{N,m} \\ \frac{dI_i^{V,m}}{dt} &= \gamma_1^m E_i^{V,m} - \gamma_2 I_i^{V,m} + \mu_i I_i^{N,m} \\ \frac{dR_i^{V,m}}{dt} &= \gamma_2 I_i^{V,m} + \mu_i R_i^{N,m} \end{cases} \tag{1}$$

where γ_1^m corresponds to the rate of loss of latency (strain dependent), γ_2 the rate of loss of infectiousness (strain independent), α_i^m the age-specific vaccine efficacy for strain m, and $\mu_i = \frac{\nu_i}{S_i^N + \sum_m (E_i^{N,m} + I_i^{N,m} + R_i^{N,m})}$ with ν_i the rate of immunisation in age group i.

We expressed the age and strain specific force of infection for age group i and strain m, λ_i^m, as follows,

$$\lambda_i^m = q_m \sigma_i^m \sum_j \sum_X c_{ij} \frac{I_j^{X,m}}{n_j},$$

where q_m represents the transmissibility parameter for strain m (imputed from the contact structure, recovery rate, and the strain-specific basic reproduction number), σ_i^m the susceptibility of age group i towards strain m, c_{ij} the frequency at which individuals in age group i make contact with those in age group j (see Section 2.3.1 for details on parameterisation), and n_j the proportion of the population in age group j.

2.3.1 Contact matrix

The structure of social contacts were taken from Fumanelli *et al.* [24], who simulated a population of synthetic individuals in order to derive frequencies of total contacts by age (in other words, the total number of contacts between individuals of age i and individuals of age j) for 26 European countries (including the United Kingdom).

Ultimately, we sought estimates of the average number of contacts a *given* person of age i had with people of aged j. Thus, we transformed the published symmetric matrix of adequate contacts in the United Kingdom ([24]) by dividing the matrix by the age structure of the population; the result was a contact matrix $c_{i,j}$ providing the average number of adequate contacts an individual of age group i has with individuals of age group j.

The United Kingdom data was stratified into yearly age groups up to the single year age bracket 89–90. With our age-structured transmission model including ages 90–100+, the following assumptions were enforced: the average number of adequate contacts individuals residing in ages 90–100+ had with individuals of age group j were set equivalent to the adequate contact quantities listed for the 89–90 age bracket ($c_{91:101,1:90} = c_{90,1:90}$); the average number of adequate

contacts individuals residing in ages 0-90 had with individuals aged 90+ equalled the average number of adequate contacts had with those aged 89–90 $c_{1:90,91:101} = c_{1:90,90}$; all single-year age bracket interactions for those aged 90+ took the value of average number of adequate contacts an individual aged 89–90 had with people aged 89–90 ($c_{91:101,91:101} = c_{90,90}$).

2.3.2 Between-season exposure history group mappings

On 1st September we perform the following updates (for clarity, the dependencies on age-group have been dropped):

- $S^N \to S^{N,h=\bar{N}}$
- $S^V \to S^{N,h=\bar{V}}$
- $\left\{E^{N,m}, I^{N,m}, R^{N,m}\right\} \to S^{N,h=m}$
- $\left\{E^{V,m}, I^{V,m}, R^{V,m}\right\} \to S^{N,h=m/\bar{V}}$

where X represents vaccination status, with N corresponding to naive/non-vaccinated and V vaccinated.

2.3.3 End of influenza season demographic processes

Although the epidemiological model ODEs were devoid of explicit demographic processes, we instead implemented fluctuations to the age structure (due to demographic processes) prior to the initiation of each influenza season.

For the time period 2010 to 2018 inclusive, we informed age-stratified population distributions for each influenza season from annual mid-year ONS population estimates [25]. Revised values for the proportion of the population contained within each unvaccinated susceptible class ($S^{N,h}$), at the outset of the influenza season, could subsequently be computed. We scaled prior influenza season values up or down as required to ensure the updated age-level proportion was matched. In addition, we assumed that, per age bracket, overall increases or decreases in population size were apportioned amongst the exposure history entities weighted according to the end-of-season exposure history proportions.

Mathematically, the entity $S_i^{N,h}$ obeyed the following,

$$S_i^{N,h} = \hat{S}_i^{N,h} \times \text{scale}_i(t+1) \quad \forall\, i \in \{1, 2, \ldots, 99, 100\}$$

where $\hat{S}_i^{N,h}$ corresponded to the start of influenza season unvaccinated susceptible class value for age class i and exposure history group h before population adjustments (i.e. demographic processes) were applied, and

$$\text{scale}_i(t+1) = \begin{cases} n_i(t+1)/n_{i-1}(t), & \text{if } i \in \{1, 2, \ldots, 98, 99\}. \\ n_i(t+1)/(n_{i-1}(t) + n_i(t)), & \text{if } i = 100. \end{cases} \tag{2}$$

3 Complementary details of the health economic modelling approach

3.1 Recovering risk group specific GP consultation rates

To permit differing propensities to consult the GP for low-risk and at-risk groups, we recover risk group specific ascertainment rates from the population level (age-stratified) ascertainment rates. Assuming a linear relationship between the risk group ascertainment rates for influenza season y and age group i,

$$\epsilon_{y,i}^{\text{At risk}} = \hat{r}\epsilon_{y,i}^{\text{Low-risk}} \qquad \text{for} \qquad \hat{r} \in \mathcal{R}^{+},$$

the ascertainment rate for the low-risk group obeys

$$\epsilon_{y,i}^{\text{Low-risk}} = \frac{\epsilon_{y,i}Z_i(y)}{Z_i^{\text{Low-risk}}(y) + \hat{r}Z_i^{\text{At-risk}}(y)} = \frac{Z_i^{+}(y)}{Z_i^{\text{Low-risk}}(y) + \hat{r}Z_i^{\text{At-risk}}(y)},$$

where $\epsilon_{y,i}$ is the risk group averaged ascertainment rate, $Z_i(y)$ is the infection case load across all risk groups in influenza season y and age group i, $Z_i^{\text{X}}(y)$ is the infection case load within risk group type X in influenza season y and age group a, and $Z_i^{+}(y)$ is the ascertained cases of age i (across all risk groups) in influenza season y.

3.2 Hospital Episode Statistics: Data extract overview

We collated HES records to inform both the likelihood of occurrence and associated cost of three classes of health outcome severity attributed to influenza: (i) outpatient attendances; (ii) emergency inpatient admissions with non-fatal outcome; (iii) emergency inpatient admission with subsequent in-hospital death.

Sections 3.2.3–3.2.6 contain supporting notes that accompanied the HES data extracts (provided by Henry Forster).

3.2.1 The data extracts

Patient records included in our analysis had a seasonal influenza ICD-10 diagnostic code (J10 or J11 code) in any diagnosis field.

Data records spanned a five year time window (September 2012 - August 2017), thus encompassing five separate influenza seasons. For each influenza season, we were provided with tables of aggregated data, comprising separate tables for at-risk and low-risk groups (for details on how risk status was determined, see Section 3.2.2).

The following age-stratified datasets were given per influenza season (with records only counted if they had primary or secondary diagnosis containing either a J10 or J11 diagnostic code):

(A) emergency admissions (including emergency re-admissions within 62 days);

(B) patients from (A) with discharge method of 'died';

(C) attended outpatient appointments within 62 days of an emergency admissions (with primary or secondary diagnosis of either J10 or J11) and the source of referral was from the same consultant as a result of an emergency admission.

Non-fatal admissions were recovered through subtracting cases with fatal outcomes (from dataset (B)) from the emergency admission data (dataset (A)).

3.2.2 Determining risk status

Records included in our analysis were assigned at-risk or low-risk group status based on a collection of risk group related ICD-10 codes. The list of ICD-10 codes used to identify patients in a risk group (provided in Table B) matched Table S10 of Cromer *et al.* [26]).

Table B. ICD-10 codes used to identify risk groups. Where a two digit code is given this includes all ICD-10 codes beginning with that code. Table reproduced from Supporting Table S10, Cromer *et al.* [26].

Condition	ICD-10 codes used
Chronic respiratory disease	J4, J6, J7, J8, Q30, J31, Q32, Q33Q34, Q35, Q36, Q37
Chronic heart disease	I05, I06, I07, I08, I09, I11, I12, I13, I20, I21, I22, I25, I27, I28, I3, I40, I41, I42, I43, I44, I45, I47, I48, I49, I5, I6, Q2
Chronic kidney disease	N0, N11, N12, N14, N15, N16, N18, N19, N25, Q60, Q61
Chronic liver disease	K7, P78.8, Q44
Chronic Neurological disease	G1, G2, G3, G4, G5, G6, G7, G8, G9
Diabetes	E10, E11, E12, E13, E14, E24, G59.0, G63.2, G73.0, G99.0, N08.3, O24, P70.0, P70.1, P70.2
Immunosuppression	Malignancies affecting the immune system: All C-codes and D37, D3[illegible]<br>HIV: B20, B21, B22, B23, B24<br>Transplantations: Z94, Z85 (Bone marrow transplants: Z94.8)<br>Conditions affecting the immune system: D56.1, D57.8, D57.0, D57.[illegible] D71, D72, D73, D76, D80, D81, D82, D83, D84, 1, K90.0
Asplenia or dysfunction of the spleen	D73, D56.1, D57.8, D57.0, D57.1, K90.0
Cochlear implants	Z96.1
Cerebrospinal fluid leaks	G96.0

3.2.3 Emergency admissions

Inpatient admission clarification:
For inpatient admissions, all extracts were limited to emergency admissions only, thus excluded day case, overnight [ordinary] elective inpatients, regular attenders and maternity admissions

We adopted the approach of solely considering the emergency admission subset of inpatient admissions because there are only two scenarios where an admission for primary diagnosis of influenza would not be classified as an emergency, with it being unexpected that influenza admissions would occur under these other admission methods.: (i) data quality/coding issue with a patient admission recorded as elective (planned) instead of emergency; (ii) an admitted patient contracts influenza in hospital which leads to cancellation of planned procedure.

Emergency admission data - Supporting notes
Emergency admission costs were based on application of 2016/17 non-elective (where the patient's admission is not planned) tariff [27].

All years were grouped to have the same Healthcare Resource Group (HRG; standard groupings of clinically similar treatments which use common levels of healthcare resources) except April 2017 to August 2017. For simplicity, a flat cost was applied for these patients to avoid complicated mapping or regrouping of data.

Where appropriate, we applied the short stay emergency adjustment to prices. In brief, this implements a percentage reduction on the main tariff to reflect the absence of a procedure [27].

We treated emergency inpatient admission events that had non-fatal outcomes (recorded discharge method other than death) separately from emergency inpatient admissions with death recorded as the discharge method.

3.2.4 Death events

Quantities acquired by counting emergency inpatient admissions with an ICD-10 J10 or J11 diagnosis code (in any position) extracted from the HES database with death recorded as the discharge method.

Within eligible records, all records logging death as the discharge method were included, in contrast with prior work by Cromer *et al.* [26], in which only records recording a discharge method of death that occurred within 30 days of admission were included in the analysis.

3.2.5 Outpatient attendance

Outpatient episode counts were limited to 'attended' outpatient appointments.

Outpatient prices were applied by relevant Treatment Function Code (TFC) and attendance type (first/follow-up attendance). In the small number of cases where there was no outpatient tariff price, the tariff price for General Medicine was applied which accounts for roughly 50% of activity within output C.

TFC clarification: TFC is a unique identifier for a treatment function. The recorded TFC reports the specialised service within which the patient is treated. TFC is based on a main speciality but also includes approved sub-specialities and treatment specialities used by lead care professionals including consultants [28].

3.2.6 Omitted cost elements

Two elements of the national tariff were not applied: (i) market force factor; (ii) long stay payments for inpatients. Further information on each cost element is provided below.

The **Market Forces Factor (MFF)** is an estimate of unavoidable cost differences between health care providers, based on their geographical location. The MFF is used to adjust resource allocations in the NHS in proportion to these cost differences, so that patients are neither advantaged nor disadvantaged by the relative level of unavoidable costs in different parts of the country. Each NHS organisation is assigned an individual MFF value. The relative values of all organisations are expressed in two indices: (i) the underlying index, which is used to adjust funding flows: in higher cost areas, commissioners receive higher levels of funding through the allocation formula so that they are able to meet the higher costs of providers for the same level of health care; (ii) the payment index, which is used in the national tariff to adjust prices at the local level for each provider.

The **long stay payment** (sometimes referred to as an 'excess bed day payment') is an additional reimbursement to the national price for patients that remain in hospital beyond an expected length of stay for clinical reasons. A long stay payment on a daily rate basis applies to all HRGs where the length of stay of the spell exceeds a specified trim point specific to the HRG and point of delivery. Specifically, the trip point is defined as the upper quartile length of stay for the HRG plus 1.5 times the interquartile range of length of stay [27].

3.3 Clinical outcome assumptions by influenza type

With the HES records having no associated influenza type information, we implemented the following assumptions to account for differences in influenza A and influenza B disease severity, discerned from prior statistical modelling studies. For a full listing of hospitalisation case rates for influenza A and influenza B, see Tables C and D.

Hospitalisation estimates:
We assumed all emergency admissions for those aged 10 years and above were due to influenza A.

This assumption was based upon prior work by Cromer *et al.* [26], in particular Table 4 demonstrating low Influenza B attributable hospitalisation rates for the age bracket 5–14 years upward.

Mortality estimates (relative to single GP consultation):
We assumed all influenza attributed in-hospital deaths are attributable to influenza A. Equivalently, we attributed zero mortality to influenza B as a simplification of prior work finding very low rates of mortality resulting from influenza B infection:

- Green *et al.* [29]: No significant influenza B attributable mortality was detected by influenza season, cause or age group.
- Cromer *et al.* [26]: Majority of influenza B attributable hospitalisation rates being within infant age groups. Therefore, in-hospital deaths due to influenza B would only be possible from the very young, which we would conjecture to be a rare outcome based on conclusions from Green *et al.* [29].
- Modelling estimate from Matias *et al.* [30]: Few influenza-attributable respiratory deaths were reported in children and adolescents <18 years. Overall, influenza-attributed respiratory deaths were primarily assigned as being caused by influenza A, rather than influenza B.

Table C. Health episode type likelihood relative to GP consultations for influenza A cases. All values are given to 2sf.

	Low-risk			At-risk		
**Age (yrs)**	**Outpatient**	**Inpatient (Non-fatal)**	**Inpatient (Fatal)**	**Outpatient**	**Inpatient (Non-fatal)**	**Inpatient (Fatal)**
**0–1**	0.065	0.79	0.0020	1.2	4.2	0.20
**2–5**	0.00069	0.13	− − −	0.077	0.88	0.028
**6–9**	0.00090	0.056	− − −	0.017	0.34	0.0094
**10–19**	0.0033	0.059	0.00016	0.0018	0.18	0.0038
**20–29**	0.0034	0.091	0.00011	0.018	0.19	0.0034
**30–39**	0.0019	0.074	8.8×10^{-5}	0.022	0.22	0.0065
**40–49**	0.0033	0.058	0.00046	0.016	0.27	0.016
**50–59**	0.0023	0.065	0.00051	0.14	0.33	0.023
**60–64**	0.0047	0.061	0.0015	0.045	0.36	0.033
**65–74**	0.0079	0.11	0.0021	0.032	0.74	0.082
**75–84**	0.012	0.31	0.013	0.048	1.0	0.14
**85+**	0.012	1.3	0.13	0.059	1.5	0.33
**All ages**	0.0041	0.0092	0.00096	0.049	0.45	0.046

− − − denotes fields with zero cases in the HES data.

Table D. Health episode type likelihood relative to GP consultations for influenza B cases. All values are given to 2sf.

	Low-risk			At-risk		
**Age (yrs)**	**Outpatient**	**Inpatient (Non-fatal)**	**Inpatient (Fatal)**	**Outpatient**	**Inpatient (Non-fatal)**	**Inpatient (Fatal)**
**0–1**	0.061	0.81	N/A	1.7	3.5	N/A
**2–5**	0.00075	0.13	N/A	0.083	0.85	N/A
**6–9**	0.000107	0.051	N/A	0.021	0.32	N/A
**10–19**	N/A	N/A	N/A	N/A	N/A	N/A
**20–29**	N/A	N/A	N/A	N/A	N/A	N/A
**30–39**	N/A	N/A	N/A	N/A	N/A	N/A
**40–49**	N/A	N/A	N/A	N/A	N/A	N/A
**50–59**	N/A	N/A	N/A	N/A	N/A	N/A
**60–64**	N/A	N/A	N/A	N/A	N/A	N/A
**65–74**	N/A	N/A	N/A	N/A	N/A	N/A
**75–84**	N/A	N/A	N/A	N/A	N/A	N/A
**85+**	N/A	N/A	N/A	N/A	N/A	N/A
**All ages**	0.0010	0.021	N/A	0.0050	0.022	N/A

N/A corresponds to ages for which the ascribed condition is assumed not to occur.

3.4 Clinical monetary costs by influenza type

We present in Tables E and F average costs associated with outpatient admissions, inpatient admissions (non-fatal) and inpatient admissions with subsequent death. These average costs are based on age- and risk-stratified hospitalisation case costs from HES records, covering September 2012 to August 2017 inclusive.

We used lognormal distributions to capture uncertainty in hospital associated health episode costs. We set standard deviations of each lognormal distribution so the ratio of standard deviation to mean values were 0.2246 (matching the standard deviation:mean value ratio for GP consultation cost).

Table E. Average costs assigned to influenza A associated hospitalisation episodes. All values given to 2dp.

	Low-risk			At-risk		
**Age (yrs)**	**Outpatient**	**Inpatient (Non-fatal)**	**Inpatient (Fatal)**	**Outpatient**	**Inpatient (Non-fatal)**	**Inpatient (Fatal)**
**0–1**	£166.86	£1342.05	£1505.75	£148.02	£2286.98	£2952.16
**2–5**	£180.34	£1203.52	– – –	£142.28	£2181.66	£2223.20
**6–9**	£119.50	£1048.85	– – –	£206.00	£2090.51	£1651.00
**10–19**	£154.05	£1125.36	£4457.00	£248.67	£1761.38	£1644.92
**20–29**	£131.62	£1079.16	£3047.50	£113.52	£1535.78	£4094.35
**30–39**	£177.40	£1225.44	£3094.00	£130.28	£1609.00	£3633.68
**40–49**	£149.01	£1311.41	£2485.10	£143.18	£1839.37	£3356.21
**50–59**	£145.29	£1359.82	£3776.56	£127.97	£1931.10	£3195.04
**60–64**	£124.77	£1466.35	£1757.13	£164.11	£2020.98	£3562.04
**65–74**	£160.83	£1495.55	£2918.46	£147.37	£1992.89	£3186.87
**75–84**	£143.00	£1652.39	£2537.00	£159.08	£2127.44	£2994.29
**85+**	£188.50	£1948.55	£2623.29	£149.96	£2168.13	£2764.05
**All ages**	£154.10	£1303.24	£2662.68	£137.58	£1978.73	£3046.46

– – – denotes fields with zero cases in the HES data.

Table F. Average costs assigned to influenza B associated hospitalisation episodes. All values given to 2dp.

	Low-risk			At-risk		
**Age (yrs)**	**Outpatient**	**Inpatient (Non-fatal)**	**Inpatient (Fatal)**	**Outpatient**	**Inpatient (Non-fatal)**	**Inpatient (Fatal)**
**0–1**	£177.35	£1330.43	N/A	£132.79	£2216.13	N/A
**2–5**	£156.84	£1182.08	N/A	£124.69	£2139.24	N/A
**6–9**	£119.50	£1047.22	N/A	£206.00	£2137.56	N/A
**10–19**	N/A	N/A	N/A	N/A	N/A	N/A
**20–29**	N/A	N/A	N/A	N/A	N/A	N/A
**30–39**	N/A	N/A	N/A	N/A	N/A	N/A
**40–49**	N/A	N/A	N/A	N/A	N/A	N/A
**50–59**	N/A	N/A	N/A	N/A	N/A	N/A
**60–64**	N/A	N/A	N/A	N/A	N/A	N/A
**65–74**	N/A	N/A	N/A	N/A	N/A	N/A
**75–84**	N/A	N/A	N/A	N/A	N/A	N/A
**85+**	N/A	N/A	N/A	N/A	N/A	N/A
**All ages**	£176.42	£1260.74	N/A	£135.36	£2165.45	N/A

N/A corresponds to ages for which the ascribed condition is assumed not to occur.

3.5 Fatal influenza case QALY loss calculation

The loss of quality adjusted life years (QALYs) from fatal cases was based on the quality adjusted life expectancy by age, discounted by some factor per annum, and adjusted for the relative life-expectancy of individuals:

$$\text{Fatal case QALY loss} = \sum_{y} \left(\sum_{a=0}^{100} D(y,a) \times E(a) \times \left(\frac{1}{1+d} \right)^{(y-y_{\text{start}})} \right)$$

whereby $D(y,a)$ is the number of deaths in simulation year y in age group a, d is the discount rate, $E(a)$ is the discounted quality adjusted value of the remaining life expectancy $L(a)$ of individuals at age a: $E(a) = \sum_{i=1}^{L(a)} \frac{Q_w(a+i)}{(1+d)^i}$ (the values of $L(a)$ are rounded to full years), with $Q_w(a)$ denoting the age-specific quality of life weight at age a.

In the absence of data imparting risk-group specific remaining life expectancy estimates, we assumed differing risk groups to have identical remaining life expectancy $L(a)$.

For parameterising the age-specific quality of life weights, Q_w, we obtained age-specific EQ-5D index population norms estimates for England from two literature sources. We took childhood estimates (which we assumed to cover 0–17 years of age) from Table 3 of [31], and values for those aged 18+ years old were sourced from Table 3.6 of Janssen and Szende [32]. A complete listing of age-specific quality of life weights values by age is presented in Table G.

Table G. EQ-5D index population norms for England. Values gleamed from Kwon *et al.* [31], ages 0–17, and Janssen and Szende [32], ages 18+.

Age group (yrs)	EQ-5D index population norms scale
<18	0.948
18–24	0.929
25–34	0.919
35–44	0.893
45–54	0.855
55–64	0.810
65–74	0.773
75+	0.703

4 Additional results

4.1 Model fitting

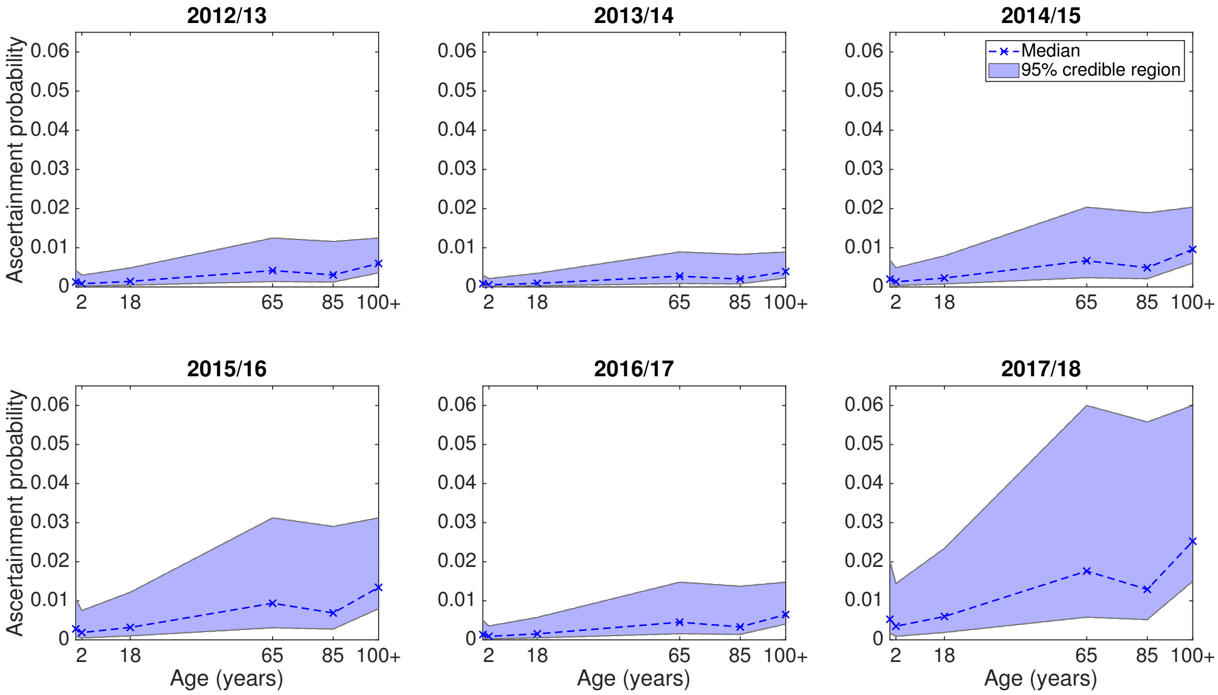

Fig E. Influenza season-specific ascertainment probabilities against year of age. Each panel corresponds to an individual influenza season, as signified by the panel title. Dashed blue lines correspond to the median profile. Shaded light blue regions illustrate the 95% credible region. Ascertainment rates were highest in the 2017/18 influenza season. Comparing across ages, there was minor variation in for those aged 0–18, with a peak at 65 years of age, a small decline up to 85 year of age, followed by increases as age rises towards 100+.

4.2 Model goodness-of-fit

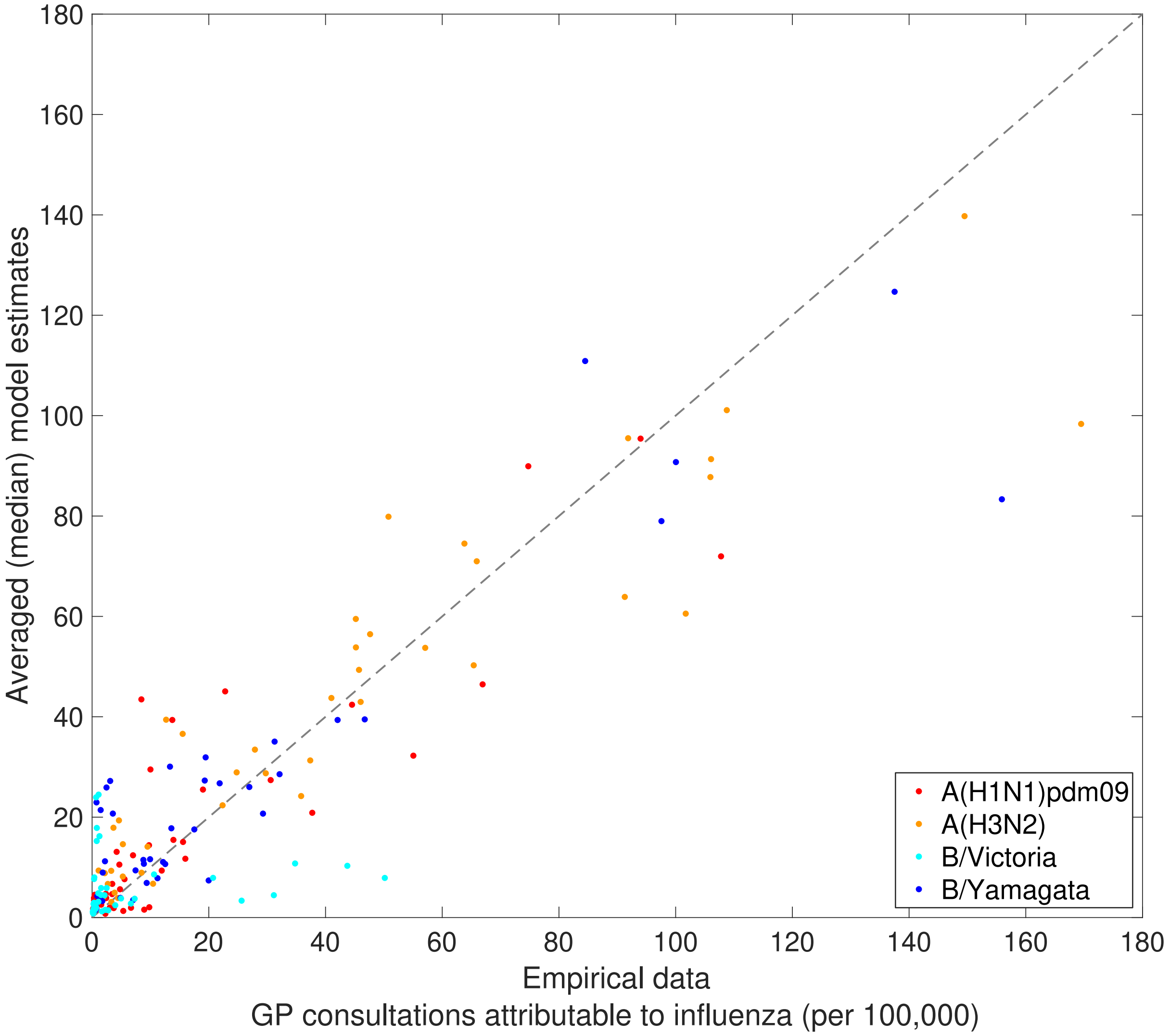

Fig F. Empirical observations against model averaged forecasts for GP consultations attributable to influenza (per 100,000 population). We display observations against median averaged forecast results, with a point per combination of influenza season, age grouping (0-1yrs, 2-10yrs, 11-17yrs, 18-40yrs, 41-64yrs, 65-84yrs, 85+yrs) and virus type. Marker colour corresponds to the virus strain applicable to that data point; red shading denoting the A(H1N1)pdm09 subtype, orange shading the A(H3N2) subtype, cyan shading the B/Victoria lineage, dark blue shading the B/Yamagata lineage. The diagonal line corresponds to a perfect correspondence between the observed data and the model forecast. We found a reasonable degree of correlation, being able to predict the general pattern of high and low observations.

4.3 Simulating vaccination programmes

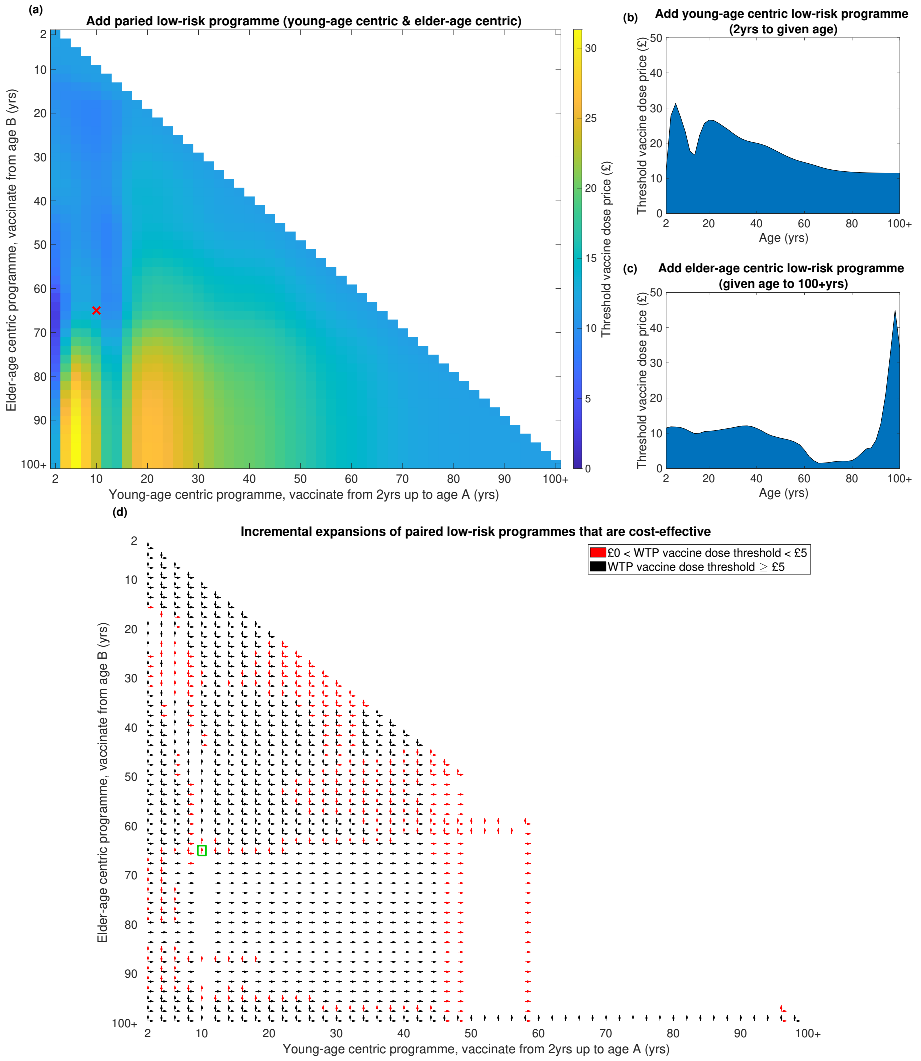

Fig G. Threshold price per vaccine dose for additional vaccines required to add a low-risk vaccine programme (relative to an at-risk only vaccination programme), using a single simulation with the most likely model parameter values. Threshold vaccine dose prices reflect the cost at a WTP of £20,000 per QALY, with 3.5% discounting for monetary costs and health effects. In all strategies we assumed 10% vaccine wastage and an administration fee per deployed vaccine of £10. **(a)** Paired low-risk programmes (combined young-age centric and elder-age centric coverage). Shading transitioning from dark shading to light shading depicts shifts from low to high prices. The white region corresponds to omitted strategies, where young-age centric and elder-age centric coverage would overlap. The red cross marks the coverage offered by the current seasonal influenza vaccine policy for England. **(b)** Addition of a low-risk young-age centric programme only. **(c)** Addition of a low-risk elder-age centric programme only. **(d)** Cost-effectiveness assessments of incrementally expanding paired low-risk vaccine programmes. Vertical arrows denote incremental expansions of the elder-age centric component (e.g. going from 70-100+yrs to 68-100+yrs) of a paired vaccination programme that were evaluated as cost-effective. Similarly, horizontal arrows denote incremental expansions of the young-age centric component (e.g. going from 2-10yrs to 2-12yrs) of a paired vaccination programme that were evaluated as cost-effective. The colour of the arrow distinguishes willingness to pay values per vaccine dose that were less than £5 (red) or £5 and above (black). The green box contains the assessment of incremental expansion from the current low-risk coverage offered by the seasonal influenza vaccine policy for England.

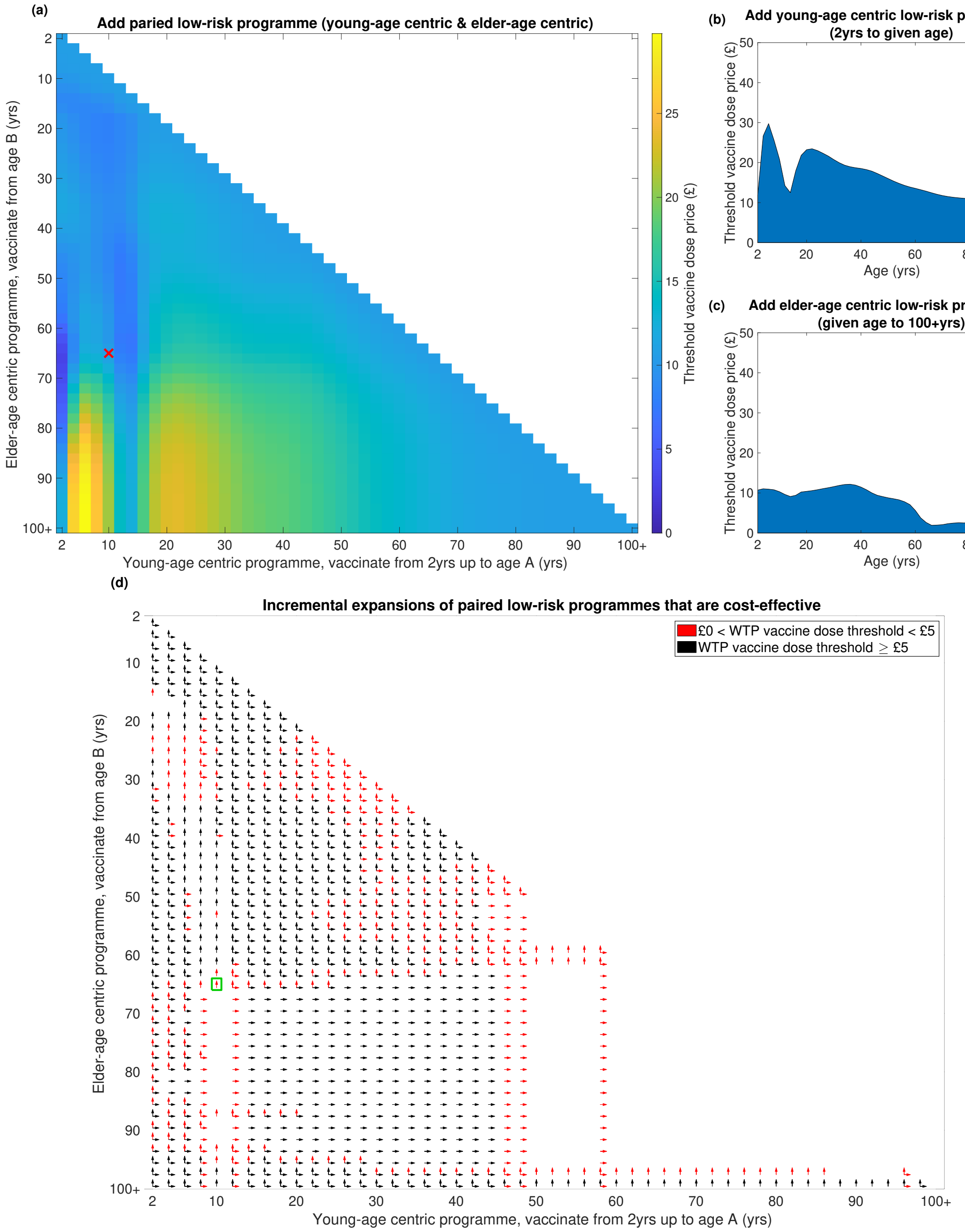

Fig H. Threshold price per vaccine dose for additional vaccines required to add a low-risk vaccine programme (relative to an at-risk only vaccination programme), using a single simulation with the most likely model parameter values and a reduced discounting rate. Threshold vaccine dose prices reflect the cost at a WTP of £20,000 per QALY, with 1.5% discounting for monetary costs and health effects. In all strategies we assumed 10% vaccine wastage and an administration fee per deployed vaccine of £10. **(a)** Paired low-risk programmes (combined young-age centric and elder-age centric coverage). Shading transitioning from dark shading to light shading depicts shifts from low to high prices. The white region corresponds to omitted strategies, where young-age centric and elder-age centric coverage would overlap. The red cross marks the coverage offered by the current seasonal influenza vaccine policy for England. **(b)** Addition of a low-risk young-age centric programme only. **(c)** Addition of a low-risk elder-age centric programme only. **(d)** Cost-effectiveness assessments of incrementally expanding paired low-risk vaccine programmes. Vertical arrows denote incremental expansions of the elder-age centric component (e.g. going from 70-100+yrs to 68-100+yrs) of a paired vaccination programme that were evaluated as cost-effective. Similarly, horizontal arrows denote incremental expansions of the young-age centric component (e.g. going from 2-10yrs to 2-12yrs) of a paired vaccination programme that were evaluated as cost-effective. The colour of the arrow distinguishes willingness to pay values per vaccine dose that were less than £5 (red) or £5 and above (black). The green box contains the assessment of incremental expansion from the current low-risk coverage offered by the seasonal influenza vaccine policy for England.

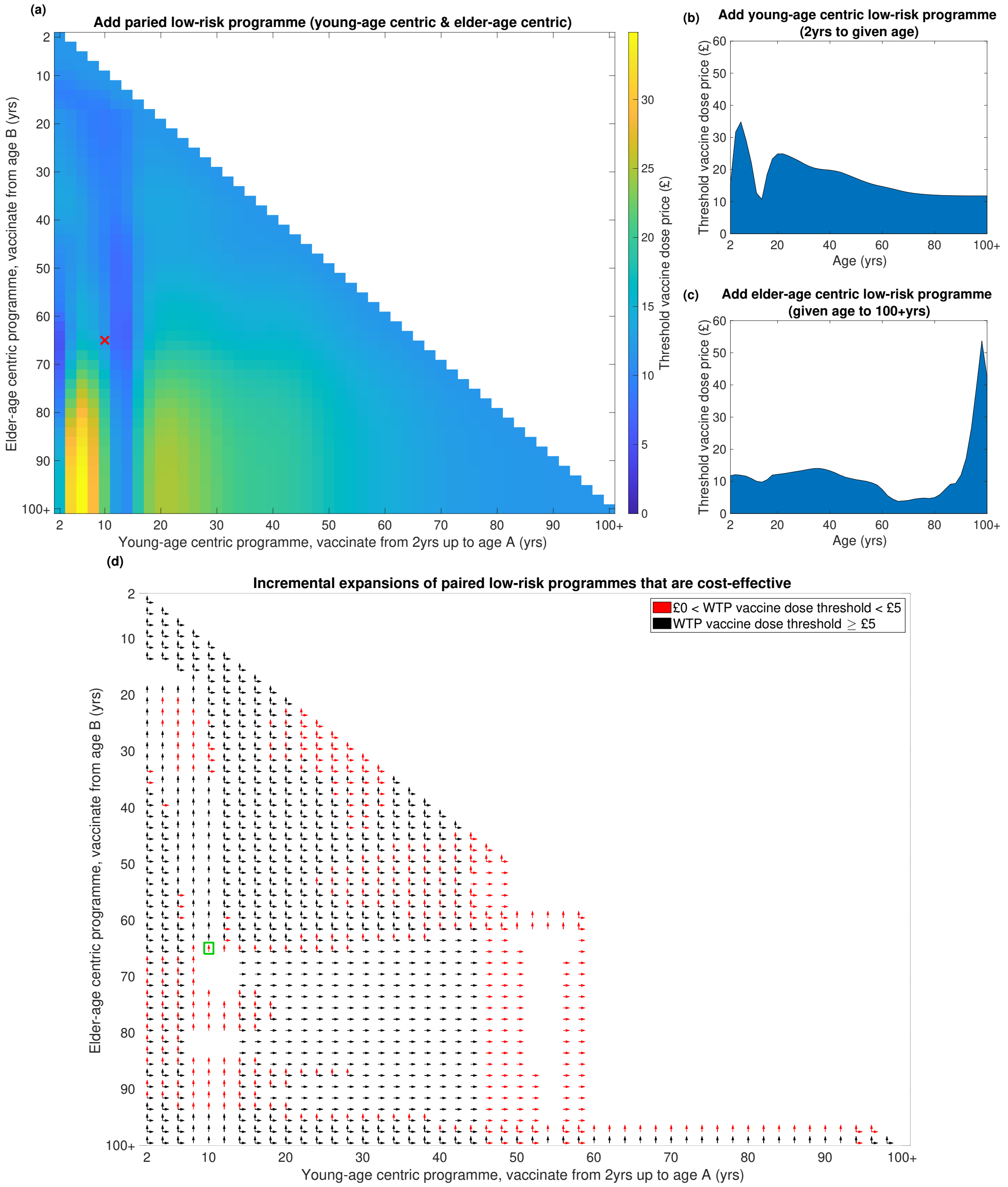

Fig I. Threshold price per vaccine dose for additional vaccines required to add a low-risk vaccine programme (relative to an at-risk only vaccination programme), under a reduced discounting rate. Threshold vaccine dose prices reflect the cost at which 90% of all simulations generate cost-effective results at a WTP of £30,000 per QALY, with 1.5% discounting for monetary costs and health effects. In all strategies we assumed 10% vaccine wastage and an administration fee per deployed vaccine of £10. **(a)** Paired low-risk programmes (combined young-age centric and elder-age centric coverage). Shading transitioning from dark shading to light shading depicts shifts from low to high prices. The white region corresponds to omitted strategies, where young-age centric and elder-age centric coverage would overlap. The red cross marks the coverage offered by the current seasonal influenza vaccine policy for England. **(b)** Addition of a low-risk young-age centric programme only. **(c)** Addition of a low-risk elder-age centric programme only. **(d)** Cost-effectiveness assessments of incrementally expanding paired low-risk vaccine programmes. Vertical arrows denote incremental expansions of the elder-age centric component (e.g. going from 70-100+yrs to 68-100+yrs) of a paired vaccination programme that were evaluated as cost-effective. Similarly, horizontal arrows denote incremental expansions of the young-age centric component (e.g. going from 2-10yrs to 2-12yrs) of a paired vaccination programme that were evaluated as cost-effective. The colour of the arrow distinguishes willingness to pay values per vaccine dose that were less than £5 (red) or £5 and above (black). The green box contains the assessment of incremental expansion from the current low-risk coverage offered by the seasonal influenza vaccine policy for England.

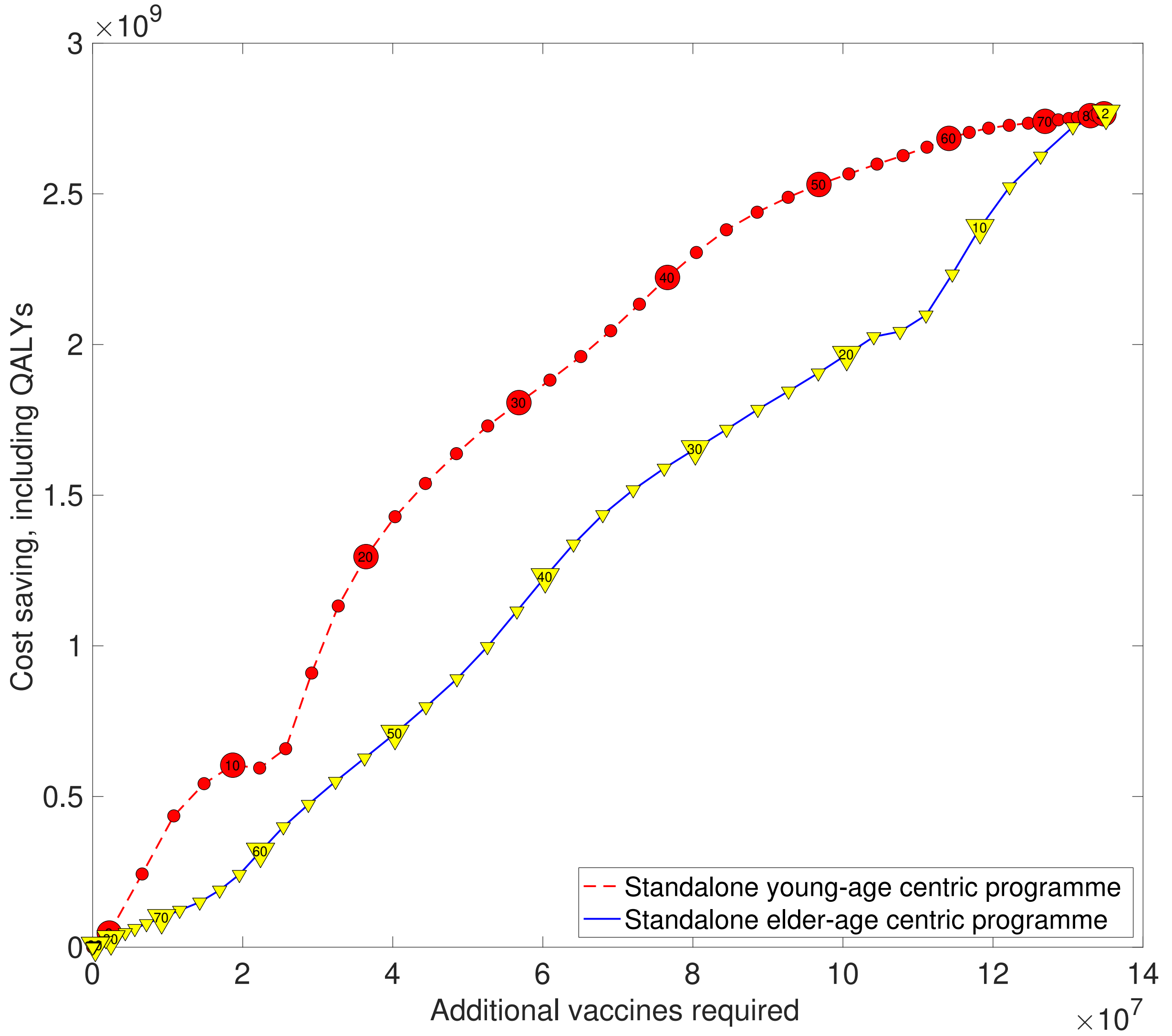

Fig J. Estimated savings against additional administered vaccines for alternative vaccine programmes (relative to an at-risk only vaccination programme). The plotted points represent median estimates for a single vaccine programme, with one QALY valued at £20,000. The red dashed line with circle markers corresponds to young-age centric strategies. Numerical values contained within the circle markers state the upper bound of age coverage represented by that particular young-age centric strategy (e.g. the marker containing "20" represents a strategy covering those between 2 to 20 years of age). The blue solid line with inverted yellow triangle markers corresponds to elder-age centric strategies. Numerical values contained within the inverted triangle markers express the lower bound of age coverage represented by that particular elder-age centric strategy (e.g. the marker containing "20" represents a strategy covering those aged 20 years old and above). The results qualitatively resemble outcomes acquired under a valuation of £30,000 per QALY.

References

1. Correa A, Hinton W, McGovern A, van Vlymen J, Yonova I, *et al.* Royal College of General Practitioners Research and Surveillance Centre (RCGP RSC) sentinel network: a cohort profile. *BMJ Open* **6**(4):e011092 (2016). doi:10.1136/bmjopen-2016-011092.
2. de Lusignan S. Codes, classifications, terminologies and nomenclatures: definition, development and application in practice. *Inform. Prim. Care* **13**(1):65–70 (2005).
3. de Lusignan S, van Weel C. The use of routinely collected computer data for research in primary care: opportunities and challenges. *Fam. Pract.* **23**(2):253–263 (2006). doi:10.1093/fampra/cmi106.
4. De Lusignan S. Effective pseudonymisation and explicit statements of public interest to ensure the benefits of sharing health data for research, quality improvement and health service management outweigh the risks. *J. Innov. Heal. Informatics* **21**(2):61–63 (2014). doi:10.14236/jhi.v21i2.68.
5. Health Protection Agency. Surveillance of influenza and other respiratory viruses in the UK publications (2014). URL https://webarchive.nationalarchives.gov.uk/20140629102650/http://www.hpa.org.uk/Publications/InfectiousDiseases/Influenza/. [Online] (Accessed: 28 September 2020).
6. Public Health England. Official Statistics: Annual flu reports (2019). URL https://www.gov.uk/government/statistics/annual-flu-reports. [Online] (Accessed: 28 September 2020).
7. Public Health England. Collection: Weekly national flu reports (2020). URL https://www.gov.uk/government/collections/weekly-national-flu-reports. [Online] (Accessed: 28 September 2020).
8. World Health Organisation. FluNet. (2019). URL http://www.who.int/influenza/gisrs_laboratory/flunet/en/. [Online] (Accessed: 28 September 2020).
9. World Health Organisation. Global Epidemiological Surveillance Standards for Influenza. (2013). URL http://www.who.int/influenza/resources/documents/WHO_Epidemiological_Influenza_Surveillance_Standards_2014.pdf. [Online] (Accessed: 228 September 2020).
10. World Health Organisation. Manual for the laboratory diagnosis and virological surveillance of influenza. (2011). URL https://apps.who.int/iris/bitstream/handle/10665/44518/9789241548090_eng.pdf?sequence=1. [Online] (Accessed: 28 September 2020).
11. Department of Health. Pandemic H1N1 (Swine) Influenza Vaccine Uptake amongst Patient Groups in Primary Care in England (2010). URL https://www.gov.uk/government/uploads/system/uploads/attachment_data/file/215977/dh_121014.pdf. [Online] (Accessed: 28 September 2020).
12. Hardelid P, Fleming DM, McMenamin J, Andrews N, Robertson C, *et al.* Effectiveness of pandemic and seasonal influenza vaccine in preventing pandemic influenza A(H1N1)2009 infection in England and Scotland 2009-2010. *Eurosurveillance* **16**(2):pii=19763 (2011). doi:10.2807/ese.16.02.19763-en.
13. Pebody RG, Andrews N, Fleming DM, McMenamin J, Cottrell S, *et al.* Age-specific vaccine effectiveness of seasonal 2010/2011 and pandemic influenza A(H1N1) 2009 vaccines in preventing influenza in the United Kingdom. *Epidemiol. Infect.* **141**(3):620–630 (2013). doi:10.1017/S0950268812001148.
14. Pebody RG, Andrews N, McMenamin J, Durnall H, Ellis J, *et al.* Vaccine effectiveness of 2011/12 trivalent seasonal influenza vaccine in preventing laboratory-confirmed influenza in primary care in the United Kingdom: evidence of waning intra-seasonal protection. *Eurosurveillance* **18**(5):20389 (2013). doi:10.2807/ese.18.05.20389-en.
15. Andrews N, McMenamin J, Durnall H, Ellis J, Lackenby A, *et al.* Effectiveness of trivalent

seasonal influenza vaccine in preventing laboratory-confirmed influenza in primary care in the United Kingdom: 2012/13 end of season results. *Eurosurveillance* **19**(27):20851 (2014). doi:10.2807/1560-7917.ES2014.19.27.20851.
16. Pebody R, Warburton F, Andrews N, Ellis J, von Wissmann B, *et al.* Effectiveness of seasonal influenza vaccine in preventing laboratory-confirmed influenza in primary care in the United Kingdom: 2014/15 end of season results. *Eurosurveillance* **20**(36):30013 (2015). doi:10.2807/1560-7917.ES.2015.20.36.30013.
17. Pebody R, Warburton F, Ellis J, Andrews N, Potts A, *et al.* Effectiveness of seasonal influenza vaccine for adults and children in preventing laboratory-confirmed influenza in primary care in the United Kingdom: 2015/16 end-of-season results. *Eurosurveillance* **21**(38):30348 (2016). doi:10.2807/1560-7917.ES.2016.21.38.30348.
18. Pebody R, Warburton F, Ellis J, Andrews N, Potts A, *et al.* End-of-season influenza vaccine effectiveness in adults and children, United Kingdom, 2016/17. *Eurosurveillance* **22**(44):pii=17–00306 (2017). doi:10.2807/1560-7917.ES.2017.22.44.17-00306.
19. Public Health England. Surveillance of influenza and other respiratory viruses in the United Kingdom: Winter 2013/14 (2014). URL https://assets.publishing.service.gov.uk/government/uploads/system/uploads/attachment_data/file/325203/Flu_annual_report_June_2014.pdf. [Online] (Accessed: 28 September 2020).
20. Public Health England. Influenza vaccine effectiveness (VE) in adults and children in primary care in the United Kingdom (UK): provisional end-of-season results 2017-18 (2018). URL https://assets.publishing.service.gov.uk/government/uploads/system/uploads/attachment_data/file/779474/Influenza_vaccine_effectiveness_in_primary_care_2017_2018.pdf. [Online] (Accessed: 28 September 2020).
21. Pebody R, Djennad A, Ellis J, Andrews N, Marques DFP, *et al.* End of season influenza vaccine effectiveness in adults and children in the United Kingdom in 2017/18. *Eurosurveillance* **24**(31) (2019). doi:10.2807/1560-7917.ES.2019.24.31.1800488.
22. Baguelin M, Flasche S, Camacho A, Demiris N, Miller E, *et al.* Assessing Optimal Target Populations for Influenza Vaccination Programmes: An Evidence Synthesis and Modelling Study. *PLoS Med.* **10**(10):e1001527 (2013). doi:10.1371/journal.pmed.1001527.
23. Hill EM, Petrou S, de Lusignan S, Yonova I, Keeling MJ. Seasonal influenza: Modelling approaches to capture immunity propagation. *PLOS Comput. Biol.* **15**(10):e1007096 (2019). doi:10.1371/journal.pcbi.1007096.
24. Fumanelli L, Ajelli M, Manfredi P, Vespignani A, Merler S. Inferring the Structure of Social Contacts from Demographic Data in the Analysis of Infectious Diseases Spread. *PLoS Comput. Biol.* **8**(9):e1002673 (2012). doi:10.1371/journal.pcbi.1002673.
25. Office for National Statistics. Dataset: Population Estimates for UK, England and Wales, Scotland and Northern Ireland (2019). URL https://www.ons.gov.uk/peoplepopulationandcommunity/populationandmigration/populationestimates/datasets/populationestimatesforukenglandandwalesscotlandandnorthernireland. [Online] (Accessed: 28 September 2020).
26. Cromer D, van Hoek AJ, Jit M, Edmunds WJ, Fleming D, *et al.* The burden of influenza in England by age and clinical risk group: A statistical analysis to inform vaccine policy. *J. Infect.* **68**(4):363–371 (2014). doi:10.1016/j.jinf.2013.11.013.
27. NHS Improvement and NHS England. NHS National Tariff Payment System 2016/17 (2017). URL https://www.gov.uk/government/publications/nhs-national-tariff-payment-system-201617. [Online] (Accessed: 28 September 2020).
28. NHS Digital. Treatment Function Code (2018). URL https://www.datadictionary.nhs.uk/data_dictionary/attributes/t/tran/treatment_function_code_de.asp?shownav=1. [Online] (Accessed: 28 September 2020).

29. Green HK, Andrews N, Fleming D, Zambon M, Pebody R. Mortality Attributable to Influenza in England and Wales Prior to, during and after the 2009 Pandemic. *PLoS One* **8**(12):e79360 (2013). doi:10.1371/journal.pone.0079360.
30. Matias G, Taylor RJ, Haguinet F, Schuck-Paim C, Lustig RL, *et al.* Modelling estimates of age-specific influenza-related hospitalisation and mortality in the United Kingdom. *BMC Public Health* **16**:481 (2016). doi:10.1186/s12889-016-3128-4.
31. Kwon J, Kim SW, Ungar WJ, Tsiplova K, Madan J, *et al.* A Systematic Review and Meta-analysis of Childhood Health Utilities. *Med. Decis. Mak.* **38**(3):277–305 (2018). doi:10.1177/0272989X17732990.
32. Janssen B, Szende A. Population Norms for the EQ-5D. In *Self-Reported Popul. Heal. An Int. Perspect. based EQ-5D*, pages 19–30. Springer Netherlands, Dordrecht (2014). doi:10.1007/978-94-007-7596-1_3.
