# Supplementary material for: Optimising age coverage of seasonal influenza vaccination in England: A mathematical and health economic evaluation
Source: PLoS Comput Biol. 2020 Oct 6;16(10):e1008278. doi: 10.1371/journal.pcbi.1008278 (PMC7567368; doi:10.1371/journal.pcbi.1008278)
Supplement: Attachment — Submitted filename: Hilletal_Response to Reviewers.docx [file pcbi.1008278.s005.docx]

**Response to Reviewers**

**Optimising age coverage of seasonal influenza vaccination in England: A mathematical and health economic evaluation** by E. M. Hill, S. Petrou, H. Forster, S. de Lusignan, I. Yonova & M. J. Keeling.

*We are grateful for the opportunity to respond to the comments of the Reviewers, which have caused us to improve the manuscript. Herein we provide a detailed response to each suggestion in blue italics interspersed with the original comments in black font.*

*Where appropriate, at the end of a comment we state in red font and within parentheses the line numbers where that edit/addition can be found within the revised manuscript (clean copy and tracked changes copy).*

**Reviewer 1**

**Major comments:**

1) This is an impressive piece of research. The authors have extended their previous model to address policy relevant questions about the health and economic consequences of expanding seasonal influenza vaccination by age groups. This is an important contribution to the literature.

*2)* A major challenge for the authors is to determine where to focus their attention given the number of reportable results included in the paper. They have focused their main conclusions on health and economic consequences of competing vaccination policies, but there are many important results which the model provides some additional insight about, including inference on strain specific reproductive numbers and strain-specific partial immunity afforded by previous infections. The following comments in this section reflect my concern that the complexity of the model and richness of the results, also means that readers will have numerous questions about the importance of underlying assumptions for the conclusions being drawn (even if these are not featured as key conclusions), not all of which can probably be reasonably subjected to sensitivity analyses. I would suggest the authors may better help a reader understand which of these types of assumptions are most critical for driving the conclusions that they draw.

***Authors’ response:***

*We thank the reviewer for the assessment of the research article and the guiding points that follow, which have helped us improve the manuscript. We outline below our revisions to address the issues raised.*

3) The model is complex and requires numerous assumptions which often not verifiable with available data and may be important for inference. Can the authors help guide the reader better about how these might affect results and how they should be viewed by readers?

***Authors’ response:***

*The reviewers remark on the model complexity necessitating several assumptions, which if relaxed possibly adjusting the outcomes, is fair. We concur that conveying these items to the reader is paramount. As a result, we have added additional sentences to express this position when introducing our transmission model:*

*“As we summarise each aspect of the framework, though the model incorporates numerous complexities we also make simplifying assumptions in order to be able to parameterise the model. We highlight these assumptions as appropriate, with potential influences on model outcomes outlined within the discussion.”*

*(Clean copy: lines 147-150)*

*(Tracked changes: lines 156-159)*

*We go on to address the specific points in turn.*

A partial list includes:

a. Assumptions needed to estimate strain specific vaccine efficacy (Table 1, Table S1).

***Authors’ response:***

*We now explicitly state how usage of a distribution of values for the vaccine efficacy, rather than simply a point estimate, would generate added variability in model projections.*

*“We recognise the use of a point estimate for a quantity with inherent uncertainty surrounding its value is a simplification. Were vaccine efficacy values to be drawn from a distribution, we would expect additional variability in model projections. However, as the point estimates reflect what was deemed as the likely vaccine efficacy given the available data, we take its usage as a reasonable assumption and its application also aided parameterisation of other facets of complexity contained in the model.”*

*(Clean copy: lines 107-111)*

*(Tracked changes: lines 115-119)*

b. Line 89: Assumption that strain distribution is maintained across age groups

***Authors’ response:***

*We have changed the text in an effort to enhance the communication of our reasoning for applying this assumption:*

*“In the absence of data breaking down how the strain composition was split between differing ages, we made a simple assumption of the strain composition being applied homogeneously across all ages. We postulate that incorporation of relevant age-stratified data would permit refinements to the parameter inference scheme to capture any heterogeneities across ages.”*

*(Clean copy: lines 84-87)*

*(Tracked changes: lines 91-95)*

*We also maintained the inclusion of a designated paragraph in the discussion to discuss prospects on incorporation of age-stratified data on strain composition.*

*(Clean copy: lines 767-773)*

*(Tracked changes: lines 817-823)*

c. Line 170: Assumptions about how strain-specific immunity afforded by vaccination carries over between seasons.

***Authors’ response:***

*We now elaborate on how our assumptions on residual immunity from vaccination are a strong generalisation, and give nods to how sufficiently detailed data may allow for alternative parameterisations to be considered.*

*“Whilst defining a relationship between two factors when sufficient data are not available to ground it on empirical observation necessitates making assumptions, we recognise a linear dependency is a strong generalisation. Thus, if there is sufficiently detailed data to underpin the models, we encourage consideration of alternative non-linear formulations for the vaccine-derived immunity propagation process. Such formulations may also permit instances of negative interference, where exposure in the prior influenza season (be that naturally or through vaccination) increases susceptibility to influenza infection during the current influenza season above the baseline value of 1.”*

*(Clean copy: lines 182-189)*

*(Tracked changes: lines 191-198)*

d. Line 202: While the motivation for assumption about average susceptibility within age bands is reasonable – any sensitivity analysis on this? Age band from 18-64 is very wide. Does this result in averaging over important heterogeneities?

***Authors’ response:***

*We thank the reviewer for putting forward this suggestion. We have added a note in the methods to caution on the possible impact of the used age bands potentially averaging over heterogeneities in those between 18 and 64 years old.*

*“We acknowledge, at this juncture, that we made a trade-off in the pursuit of model parsimony by letting the younger adults category have a notable breadth in age range, which may lead to averaging over heterogeneities.”*

*(Clean copy: lines 220-222)*

*(Tracked changes: lines 236-238)*

*The point on the age band 18-64 being wide is valid and we view exploration of this assumption as meriting extensive further investigation. Within the discussion, we add a paragraph to acknowledge as valuable directions for further study both sensitivity to this assumption and the gathering of empirical data to inform age-dependent susceptibility profiles.*

*“A second age-dependent attribute warranting continued refinement is susceptibility to infection. For purposes of model parsimony, we enforced rigidity in the permitted form of the age-dependent susceptibility profile. By using four age classes, with younger adults (18-64 years of age) mandated to have lesser susceptibility compared to all other, we may have averaged over important heterogeneities. The impact of relaxing these assumptions merits further study. In tandem, the gathering of empirical data to inform age-dependent susceptibility profiles would be immensely beneficial with regards to reducing parameter uncertainty.”*

*(Clean copy: lines 774-780)*

*(Tracked changes: lines 824-830)*

4) Lines 212-220: I am struggling to fully understand procedures for model initialization. How did authors establish age-group specific residual immunity f(h,m) at beginning of simulations?

***Authors’ response:***

*Thanks for drawing to our attention that we did not explicitly state how age-group specific residual immunity was initialised at the beginning of each simulation. No portion of the population was initialised with residual immunity. We have added this description into the text.*

*We have also combined into a single paragraph the passages of the text referring to immunity mapping between influenza season and initialising infecteds at the beginning of each influenza season.*

*(Clean copy: lines 223-234)*

*(Tracked changes: lines 239-251)*

5) Ascertainment probabilities – for those >100y which serve as reference group from which all other age group ascertainment probabilities are calculated is very low. The ascertainment probabilities seems terribly small, in the majority of cases well <1%. Is this plausible? Am I interpreting Table 6 correctly?

***Authors’ response:***

*We thank the reviewer for the remark and queries regarding the ascertainment probabilities. Yes, that interpretation of Table 6 is correct. Given uncertainty surrounding ascertainment of influenza cases, we choose to include it as a parameter to be fit within the fitting scheme. The values returned by the fitting scheme therefore signify the range of values for the ascertainment parameter that (in combination with the inferred values for the other parameters) provide the closest correspondence between our model simulations and the observed data. Naturally, these outcomes are caveated by being generated with a given disease state structure and our stated assumptions. Other choices may result in differing values for the ascertainment parameters being acquired.*

*That being said, as described within the “Observation model” subsection of the methods, the ascertainment probability captures multiple processes that can impact the likelihood of those infected by influenza being observed and the disease status correctly determined. These processes include those infected being symptomatic, consulting with a GP, being recorded as having ILI, and having a detectable influenza viral load. Each of these diminish the probability of an infected person being ascertainable, and therefore the ascertainment estimates of less than 1% in some influenza seasons could be plausible.*

6) Figure comments

a. Fig 5 (and similar figures in the supplement) are too difficult to read. In particular the arrowheads are very hard to make out.

***Authors’ response:***

*To improve readability, we have split the information for the paired strategies that was conveyed originally as a single panel (panel (a) ) into two separate panels. The figure captions have been updated to include specific details on what the arrows correspond to:*

*“(b) Addition of a low-risk young-age centric programme only. The arrows depict those incremental expansions of young-age centric low-risk vaccination programmes, with schemes covering two additional years of age (e.g. going from 2-10yrs to 2-12 yrs), that would be cost-effective (red arrows for willingness to pay values per vaccine dose less than £5, black arrows for willingness to pay values per vaccine dose above £5). (c) Addition of a low-risk elder-age centric programme only. The arrows depict those incremental expansions of elder-age centric low-risk vaccination programmes, with schemes covering two additional years of age (e.g. going from 70-100+yrs to 68-100+yrs), that would be cost-effective. (d) Cost-effectiveness assessments of incrementally expanding paired low-risk vaccine programmes. Vertical arrows denote incremental expansions of the elder-age centric component of a paired vaccination programme that were evaluated as cost-effective. Similarly, horizontal arrows denote incremental expansions of the young-age centric component of a paired vaccination programme that were evaluated as cost-effective.”*

*(Clean copy: Fig. 5)*

*(Tracked changes: Fig. 5)*

b. Fig 6 (and similar figures in supplement) – I am really struggling to interpret these! The size shapes and shapes of the figures are difficult for me to see at full size. I fear I am missing the conclusions here. Can simpler figures be made, even if more panels are required to show all the information?

***Authors’ response:***

*We regret our initial version of the figure was difficult to interpret and the intended conclusions may not be successfully drawn. With thanks to the reviewer for the suggestion, we have simplified the figure and focus on comparing sole young-age centric strategies against sole elder-age centric strategies.*

*(Clean copy: Fig. 6)*

*(Tracked changes: Fig. 6)*

7) Are the authors making their code available?

***Authors’ response:***

*We can confirm the code will be available and we have added details of the repository housing relevant files to the Data Availability statement.*

**Minor comments:**

1) Line 33: Claim that previous models guiding England policy were calibrated to pre-2009 data begs the question of whether this is materially important for the insights generated here. Of course, it is good that the authors have calibrated their model to more recent data, but this claim implicitly suggests that a model similar to theirs calibrated to older data might result in different conclusions. Is that actually true? If not, I would suggest that they remove this from the Introduction so that the main advance here remains focused on their treatment of mechanisms of carry-over immunity from season to season

***Authors’ response:***

*The reviewer raises an excellent point and we agree that it would be prudent to keep the message focused on the treatment of mechanisms of carry-over immunity from season to season. We have therefore removed the paragraph from the Introduction.*

*(Tracked changes: lines 33-37)*

2) Line 225: Claim that health outcomes only rather than epidemiological processes are dependent upon risk group membership seems too string a statement. I am not quibbling with the authors’ approach here, as indeed risk group most profoundly affects health outcome condition on infection status, but the assertion that risk group is entirely divorced from transmission dynamics is probably too strong a statement to make.

***Authors’ response:***

*We have rephrased this passage of the text in an attempt to reflect that transmission dynamics being independent of risk group status was a modelling assumption.*

*“However, given risk group status most profoundly affects health outcome condition on infection status, to lessen model complexity we assumed epidemiological processes were not dependent on risk status.”*

*(Clean copy: lines 245-247)*

*(Tracked changes: lines 262-265)*

3) Is there a good reason to retain the labels for the 5 different pediatric vaccination schemes (Prim, PrimSec, etc)? Why not just label these as the age groups targeted which is nearly as concise and is easier for readers as they don’t need to remember anything? Maybe I am missing an obvious rationale for this.

***Authors’ response:***

*We are grateful to the reviewer for expressing this potential instance of superfluous labelling. We wholeheartedly agree with the point raised. We have reverted to labelling the paediatric strategies by the age groups targeted, including modifying the x-axis labelling in Fig. 4.*

**Reviewer 2**

**Major comments:**

This manuscript has some very interesting ideas and the analysis presented is certainly very thorough. However, after reading the manuscript many times, I still don’t fully understand key points of the article:

***Authors’ response:***

*We would like to thank the reviewer for their comments and for the items they highlight. We outline below our changes in response to the stated concerns.*

1) Unless I completely misunderstood this manuscript, the authors are comparing many strategies to a “high-risk only strategy”, that would not include all people over 65 years old or all primary school aged children. However, according to this website:

<https://www.nhs.uk/conditions/vaccinations/flu-influenza-vaccine/>

People over 65 years old, children aged 2 and 3 and children in primary school are already getting the vaccine. Then it is unclear to me what the authors are testing against. Why would it make sense to use a “high-risk only” strategy as a baseline if this does not correspond to the current guidelines of the country?

If I misunderstood what you are comparing, then more explanations are needed.

If I understood this correctly, your baseline strategy is not the one currently used by the UK government. Unless there is a debate about contracting (so vaccinating less people) the status quo policy, then the article needs a very strong rationale about why this is a relevant comparison. In particular, it either needs to state very clearly why contracting the vaccination for the elderly is a valuable exercise (considering how unpopular would be to take away vaccination from older people, who are the higher-risk group for influenza and the ethical implications of doing this) or focus on studying expanding the vaccination program to age groups not currently offered vaccination.

Given the fact that the UK already offers vaccination both to primary school aged children and the elderly, then I believe that the most relevant scientific question might be if it would be beneficial to expand the current recommendations to expand the age vaccinated (either by expand the children-centric part of reducing the age to start vaccinating the elderly groups). This is already somewhat covered in the manuscript (except that the comparison is against a high-risk strategy only), but it is buried among many other results. It might be better to concentrate on this and all the related results for this (expanding children only, older age groups only, both, optimal level of age coverage for a fixed cost of seasonal influenza vaccine, threshold vaccine dose price, etc). Maybe a table/figure with which groups are covered under the baseline strategy and which ones are tested (in age bands, one obviously would not want to include the >1000 strategies tested) could be very helpful.

***Authors’ response:***

*The reviewer is correct that the current UK seasonal influenza vaccine programme offers vaccines (for free) to those aged over 65 years old, children aged 2 and 3 and in primary school, as well as all those in an “at-risk” group (irrespective of age).*

*On the matter of running strategies that contract the age coverage relative to the present-day vaccine programme, the general underlying principle to cost-effectiveness based decision-making is that the opportunity cost of adopting/expanding new interventions or strategies requires other activities to be displaced in order to fund them. This is reflected in the cost-effectiveness thresholds adopted by decision-makers, such as the National Institute for Health and Care Excellence (https://www.nice.org.uk/Media/Default/About/Who-we-are/Corporate-publications/Corporate-and-business-plans/business-plan-18-19.pdf), and methodological guidance provided to evaluators. We therefore believe that this should be reflected consistently across investment and disinvestment recommendations. As such, it is as important to consider a reduction in vaccine coverage as it is to consider an expansion - showing that one should not disinvest in the current strategy would therefore be an important result.*

*Another consideration is that policy makers may have an interest in gathering second opinions from multiple models on what are deemed as acceptable prices to pay per vaccine dose that ensure cost-effectiveness of a given vaccine strategy. Analysis of counterfactual scenarios such as “at-risk only” vaccination programmes can help provide insights on what the maximum price one would be willing to pay for those additional vaccines administered to “low risk” individuals.*

*Additionally, we have expanded the results to explicitly comment on outcomes obtained when performing model simulations using age coverages that align with the current vaccination policy. In Figure 5, we have added markers to indicate the current vaccination programme, and in the text we comment:*

*“Furthermore, the coverage offered by the current seasonal influenza vaccine policy for England to those at low-risk (young-age centric component for ages 2-10, elder-age centric component for those aged 65 and above) lay within the £10-£15 threshold vaccine dose price bracket.”*

*(Clean copy: lines 629-632)*

*(Tracked changes: lines 661-663)*

*In Figure 7, we have included two new panels that analyse the optimal age coverage for a given vaccine dose price, with the condition that the strategy must include all those aged 65 and over. The associated text reads:*

*“We saw consistent behaviour when reperforming the analysis with the addition of a condition mandating that the elder-age component had to include, at the very least, all those aged 65 and above. Across each tested fixed vaccine dose price, the discerned optimal lower bound of age coverage for the elder-age component of the programme did not adjust from 64 years of age. Imposing the mandated age coverage condition also led to a narrowing in the range of influenza vaccine dose prices for which addition of a low-risk vaccination programme would be viable.”*

*(Clean copy: lines 651-656)*

*(Tracked changes: lines 697-702)*

2) Overall, the sections regarding the implementation of the vaccination strategies are very confusing. I believe it will strongly benefit from a thorough rewrite, perhaps with more tables and figures summarizing the main points.

***Authors’ response:***

*With the endeavour of easing interpretation,* *we have undertaken a rewrite of the “Simulative vaccination strategies” subsection of the methods. Additionally, in the opening paragraphs we have introduced a broad overview of the three batches of vaccine scenarios that will be considered, before going on to describe each batch under dedicated headings.*

*(Clean copy: lines 312-339)*

*(Tracked changes: lines 332-362)*

3) I am intrigued about the value of the Prior influenza season vaccine efficacy propagation being 0. More explanation is needed. Does that mean that being vaccinated the previous year did not provide any additional protection?

***Authors’ response:***

*We thank the reviewer for the questions regarding the vaccine efficacy propagation parameters, and regret the information asked for was not adequately provided in the initial submission. We now provide examples of what xi being 0 and 1 corresponds to:*

*“If xi=0, there would be no carry over of prior influenza season vaccine efficacy and, therefore, no reduction in susceptibility (with c_{m}(y) = 1). On the other hand, if xi=1, the full efficacy of the previous influenza vaccine toward strain $m$ was retained.”*

*(Clean copy: lines 180-182)*

*(Tracked changes: lines 189-191)*

Also, did the authors allowed this parameter to take negative values? There is a big debate now in the field about the topic of negative interference. I think this parameter should be allowed to be negative.

***Authors’ response:***

*We are also grateful to the reviewer for asking this excellent question. For this piece of analysis, we did not allow for a negative interference mechanism that would allow for the start of season susceptibility towards a strain to be above 1 (i.e. exceed the standard baseline value of 1). This would be a very interesting expansion of the current framework requiring extensive investigation - and something we will consider for future work. We now include in the methods a statement to highlight the assumptions we have made regarding the vaccine-derived immunity propagation mechanism and how they can be iterated upon:*

*“Whilst defining a relationship between two factors when sufficient data are not available to ground it on empirical observation necessitates making assumptions, we recognise a linear dependency is a strong generalisation. Thus, if there is sufficiently detailed data to underpin the models, we encourage consideration of alternative non-linear formulations for the vaccine-derived immunity propagation process. Such formulations may also permit instances of negative interference, where exposure in the prior influenza season (be that naturally or through vaccination) increases susceptibility to influenza infection during the current influenza season above the baseline value of 1.”*

*(Clean copy: lines 182-189)*

*(Tracked changes: lines 191-198)*

Considering the that the authors did a cost-effectiveness analysis, it is conceivable that the vaccine would be less cost-effective if this parameter is negative, so I believe this needs to be explored/discussed.

*We put forward as part of the limitations section of the discussion how negative interference could impact the health economic analysis.*

*“Finally, we stress that the findings borne out from our cost-effectiveness analysis are estimates under a specific modelling framework and collection of assumptions. For example, compared to the circumstances presented throughout this manuscript, the presence of an effect such as negative vaccine interference in a subset of age groups could conceivably diminish the cost-effectiveness of alternate vaccine programmes. We advocate ongoing research into seasonal influenza immunity interactions across multiple influenza seasons to enhance biological understanding of seasonal influenza transmission dynamics, which will concurrently heighten the robustness of any subsequent health economic analysis.”*

*(Clean copy: lines 797-804)*

*(Tracked changes: lines 847-853)*

4) The values of the R0 obtained for the A strains are really high. How was R0 computed? I could not find that information anywhere in the article. At any given season, there is a percentage of the population that should be immune to the circulating strain, as it is indeed indicated by the authors’ estimate on the “modification to susceptibility due to natural infection”. Hence, I am wondering if the authors should compute R_effective for each season in addition to R0, since this will give a more realistic idea of what each season is experiencing in terms of transmission.

***Authors’ response:***

*We agree with the reviewer that the mechanistic effects of age-dependent susceptibility and residual immunity to prior influenza exposure would act to give an R_effective that was lower than R_0.*

*We also apologise for our error in not having included details on R_0 in the original submission. The R_0s for each strain were a collection of parameters to be fit by the inference scheme, that were subsequently used to compute the transmissibility of each influenza virus strain beta_m. This information has been added to the “Parameter fitting” subsection of the Methods:*

*“All the parameters listed above were used directly in the model, with the exception of the basic reproduction number for each influenza virus strain, R_{0_{m}}, which corresponded to the dominant eigenvalue of the next generation matrix for that particular strain. Instead, on a given iteration of the inference scheme we used R_{0_{m}} to calculate the transmissibility of each influenza virus strain, beta_m. Note that the computed value of beta_m, when used in combination with the recovery rate gamma_2 and the age-structured contact array, would generate a next generation matrix with R_{0_{m}} as its dominant eigenvalue.”*

*(Clean copy: lines 289-294)*

*(Tracked changes: lines 307-313)*

5) Goodness of fit (paragraphs l. 456-473) It seems that the only comparison between the data and the 100 simulations obtained through optimization is a visual comparison given in figure 3. I think a more quantitative assessment of the model outcomes vs data is needed. For instance, what how does the median and inter-quartiles of the simulations compare to the data? It seems that for some years and some age groups, the model fits pretty well the data (eg. 2014-2015) but not so much for others (eg 85+ for 12/13 season or 15/16) and in general it vastly overestimates either B-strain when the other one was predominant. While there is a comment in the text about the older age group and B-subtype not being fit properly, I believe a more thorough/quantitative analysis of the fit is warranted.

***Authors’ response:***

We agree that in general a goodness of fit measure between model and data is desirable. For statistical models there is a well-defined and rigorous method of assessing goodness of fit, whereas unfortunately for dynamics, mechanistic models there is no such agreed approach and this is very much an open research question. The inherent difficulty is that while statistical models are matched ‘separately’ to each data point, in most mechanistic models small errors at one time point may cascade to later time points. For example, in our model we forecast the dynamics over six influenza seasons from a set of initial conditions, therefore there are complex nonlinear dependencies between the aggregate summary statistics that we present in Figure 3.

We have attempted to address the reviewer’s concern by plotting observations against forecast results for strain, season and age-group *(Supporting Information: Fig. S6)*. This shows a reasonable degree of correlation, being able to predict the general pattern of high and low observations.

6) Over which years were the vaccination strategies computed to assess their cost effectiveness? At some point of the text (l.285) it is stated that the calibration was done from 2012-2018, at some other point the authors talk about the baseline vaccination strategy and how they defined it in 2009/10 (l. 291) and finally when the authors define the time horizon of the model they come back to 2012-2018 (l.407).

***Authors’ response:***

*We apologise for the confusion caused. There was a distinction between the time period covered by a single model simulation, and the influenza seasons then used to assess strategy cost-effectiveness. In full, while all model simulations began from the 2009/10 influenza season, it was the period 2012/13 to 2017/18 over which model outputs were used to assess cost-effectiveness. We have sought to clarify this through the following revisions in the text:*

*“All model simulation began at the start of the 2009/2010 influenza season (i.e. September 2009), though the time horizon applicable for assessing cost-effectiveness began from the 2012/13 influenza season (further details are provided in the “Cost-effectiveness analysis" subsection of the Methods). For our baseline strategy, the 2009/10 influenza season matched historical vaccine uptakes and efficacies of the pandemic influenza vaccine (for both low-risk and at-risk groups), while for the 2010/11 influenza seasons onward only vaccination of at-risk groups (matching historical uptake and efficacies) were simulated.”*

*(Clean copy: lines 323-329)*

*(Tracked changes: lines 346-352)*

*“There was a distinction between the time period covered by a single model simulation, and the time horizon used to assess strategy cost-effectiveness. For initialising the system, all model simulations began from the 2009/10 influenza season. For our cost-effectiveness assessment, we used a time horizon spanning the 2012/13 to 2017/18 influenza seasons, to coincide with the historical influenza season data the model had been fit too.”*

*(Clean copy: lines 478-482)*

*(Tracked changes: lines 508-512)*

7) I don’t quite understand how the cost-effectiveness analysis was carried out: was this a retrospective analysis of what would have been the most cost-effective program for those years? If so, I think this needs a strong rationale of why carrying out a retrospective analysis of what would have been cheaper/more effective would be important today. Were the interventions modeled in those years used as some sort of average to determine the cost of each of these alternative strategies and to decide which ones would be the more cost effective in the future? The paper as it stands is very hard to follow and I could not understand what was done.

***Authors’ response:***

*One issue, as addressed by the review above, is in knowing if (and when) the model will be a good fit to the data. An ideal approach would be to perform a fully prospective analysis, forecasting the strain dynamics, the vaccine efficacy and the cost-effectiveness of the vaccination strategy. While the first and last of these is possible within our mathematical framework, predicting the future level of vaccine efficacy is beyond the scope of this work. We have therefore taken a retrospective approach. This can either be viewed as a purely historical review of what could have been achieved, or (following the precedent from early work) we can view the past six influenza seasons as representative of the future - enabling us to consider which strategies are likely to prove cost-effective going forwards.*

*A new opening paragraph has been placed in the cost-effectiveness subsection of the methods to summarise the methodology, along with reference to other studies that have applied a similar analysis:*

*“The process to evaluate cost-effectiveness had similar characteristics to that of approaches used by Tsuzuki et al. and Baguelin et al. It involved using the reconstructed epidemic profiles under the actual (historically undertaken) vaccination programme. We then used the same model to estimate the number of infections that occurred for each influenza strain by age and risk group that would have occurred under an alternative vaccination programme. In the final step, the economic model mapped the epidemiological outcomes to clinical outcomes, providing monetary costs and health utility quantities per strategy that could be used to judge whether a proposed strategy was cost-effective compared to a baseline strategy.”*

*(Clean copy: lines 470-477) (Tracked changes: lines 500-507)*

**Minor comments:**

Cost-effectiveness should be cost-effective in l.484

***Authors’ response:***

*Revised as suggested.*

*(Clean copy: line 562)*

*(Tracked changes: lines 593)*

Lines 582-590, the text refers to figure 3 but given what figure 3 is, I believe this is not the correct figure (making the paragraph difficult to follow)

***Authors’ response:***

*We thank the reviewer for spotting the figure reference error. These have been changed to refer to figure 5.*

*(Clean copy: lines 673,677)*

*(Tracked changes: lines 720,727)*

l.433-435 “error measure value below 30,000” what does that mean? Where does the 30,000 come from? More explanation needed

***Authors’ response:***

*We stated the quantity (30,000) to reflect a threshold value for the deviance measure (Eq. 4) that was satisfied by all retained parameter sets. It was remiss of us to have not referenced the relevant equation originally within the sentence. We have amended the passage to reference the relevant equation.*

*“All 100 retained parameter sets adhered to the mandatory temporal criteria of peak infection in each influenza season occurring prior to March, and each returned a deviance measure below 30,000 (Eq. (4))”*

*(Clean copy: lines 509-511)*

*(Tracked changes: lines 539-541)*

Paragraphs starting on lines 305 and 326 are unclear and would benefit from some re-writing.

***Authors’ response:***

*With the intent of improving readability, we have revised both paragraphs.*

*For the paragraph originally beginning on line 305, please see:*

*(Clean copy: lines 353-361) (Tracked changes: lines 376-387)*

*For the paragraph originally beginning on line 326, please see:*

*(Clean copy: lines 377-384) (Tracked changes: lines 403-413)*

I wonder if there is a better way to represent the information contained in figures 5 and 6 that are very hard to understand.

***Authors’ response:***

*We have revised both Figure 5 and Figure 6 by adding in additional panels and splitting the information across them.*
